# A low-nuclear Ag_4_ nanocluster as a customized catalyst for the cyclization of propargylamine with CO_2_

**DOI:** 10.1038/s41467-023-42723-3

**Published:** 2023-11-01

**Authors:** Lin Li, Ying Lv, Hongting Sheng, Yonglei Du, Haifeng Li, Yapei Yun, Ziyi Zhang, Haizhu Yu, Manzhou Zhu

**Affiliations:** 1 Department of Chemistry and Centre for Atomic Engineering of Advanced Materials, Anhui University, Hefei, 230601 China; 2 Key Laboratory of Structure and Functional Regulation of Hybrid Materials of Ministry of Education, Hefei, 230601 China; 3 Key Laboratory of Functional Inorganic Material Chemistry of Anhui Province, Anhui University, Hefei, 230601 China; 4 Anhui Tongyuan Environment Energy Saving Co., Ltd., Hefei, 230041 China

**Keywords:** Structural properties, Homogeneous catalysis

## Abstract

The preparation of 2-Oxazolidinones using CO_2_ offers opportunities for green chemistry, but multi-site activation is difficult for most catalysts. Here, A low-nuclear Ag_4_ catalytic system is successfully customized, which solves the simultaneous activation of acetylene (-C≡C) and amino (-NH-) and realizes the cyclization of propargylamine with CO_2_ under mild conditions. As expected, the Turnover Number (TON) and Turnover Frequency (TOF) values of the Ag_4_ nanocluster (NC) are higher than most of reported catalysts. The Ag_4_* NC intermediates are isolated and confirmed their structures by Electrospray ionization (ESI) and ^1^H Nuclear Magnetic Resonance (^1^H NMR). Additionally, the key role of multiple Ag atoms revealed the feasibility and importance of low-nuclear catalysts at the atomic level, confirming the reaction pathways that are inaccessible to the Ag single-atom catalyst and Ag_2_ NC. Importantly, the nanocomposite achieves multiple recoveries and gram scale product acquisition. These results provide guidance for the design of more efficient and targeted catalytic materials.

## Abstract (web summary)

The preparation of 2-Oxazolidinones using carbon dioxide offers a productive yet clean synthesis route. Here, a low-nuclear Ag_4_ nanocluster catalytic system helps the simultaneous activation and the cyclization of propargylamine with CO_2_.

## Introduction

The conversion of CO_2_ into high-value-added chemicals^1–7^, such as starch^8^, carboxylic acid^9,10^, propylene carbonate^11,12^, and 2-oxazolidinone^13^, is considered a promising approach to achieve carbon neutrality and has become a hot topic in the field of catalysis. In particular, 2-oxazolidone compounds have important application potential in organic intermediates, antibacterial drugs and chiral auxiliaries^14,15^. Ideally, the greenest preparation of 2-oxazolone compounds is the cyclization of propargylamine with CO_2_. However, due to the unique structure of propargylamine, which contains both acetylene (-C≡C) and amino (-NH-) functional groups, it is difficult for most current catalysts to achieve this transformation^16–19^. Therefore, there is an urgent need to customize a catalyst with multiple active sites for the cyclization of propargylamine with CO_2_.

Single-atom catalysts (SACs) have been widely used for CO_2_ conversion due to their high molar utilization, clear active site, and unique electronic structure^20–23^. However, the presence of only a single metal site inherently limits SACs performance^24–28^. In contrast, low-nuclear nanoclusters (NCs) not only show the same characteristics as SACs but also benefit from synergistic effects between adjacent metals^29–36^. However, low-nuclear-weight NCs are more prone to unpredictable structural transformations under harsh environments^37,38^, making it difficult to identify the true active component. Scott et al. reported that alkyne-protected Cu_20_ NC do not require harsh pretreatment during catalysis^39^, Wang et al. reported that an alkyne-protected Au_38_ NC exhibited superior performance compared to that of a sulfate-protected Au_38_ NC^40^. Zheng et al. found that the activity of intact Au_34_Ag_28_(PhC≡C)_3_ is significantly better than that of partially or completely removed ligands^41^. Alkyne ligands, as metal-organic ligands, are considered to play an important role in improving the catalytic performance^42–44^.

Therefore, we designed a low-nuclear Ag_4_ NC protected by alkynes for the cyclization of propargylamine with CO_2_. As expected, the customized Ag_4_ NC achieved the highest TON value of 5746.2, significantly higher than that of reported catalysts and the corresponding Ag_2 _NC, Ag_6 _NC and Ag_9 _NC. Moreover, three Ag_4_ *NC intermediates were captured and confirmed their structures by ESI and ^1^H NMR. The key role of four Ag atoms revealed the feasibility and importance of low-nuclear catalysts at the atomic level. More importantly, the obtained Ag_4_/TNT nanocomposite afforded the product at the gram scale.

## Result and discussion

**Fig. 1:**
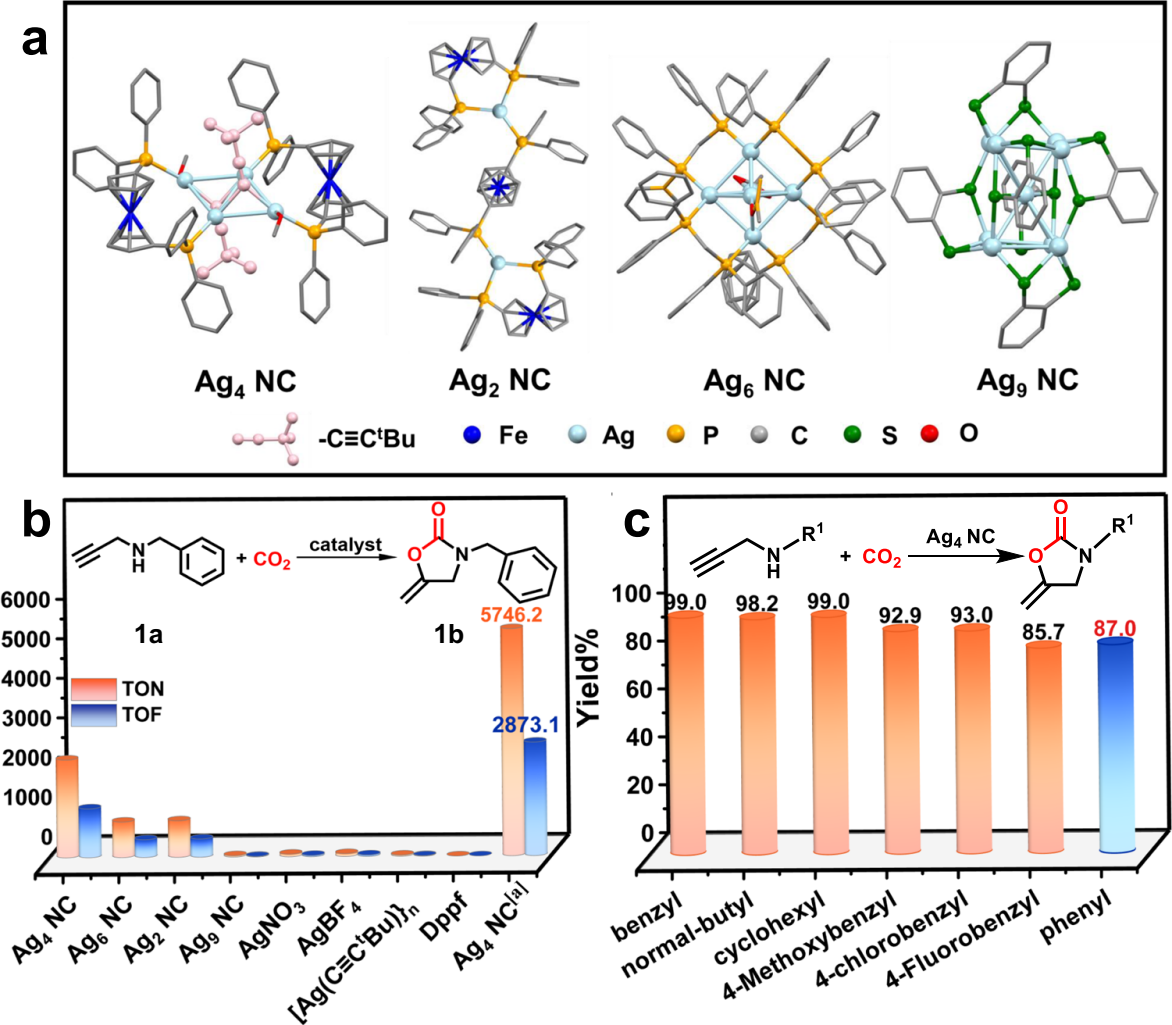
Activity comparison and substrate expansion. **a** Total structure of the Ag_n_ (*n* = 2,4,6,9) NCs. **b** TON and TOF value of different catalysts for CO_2_ cycloaddition of N-benzylprop-2-yn-1-amine. Reaction conditions: Ag_4_ NC (0.04 mol%), propargylamine (0.5 mmol), DBU (0.05 mmol), solvent (1 mL), 25 °C and CO_2_ balloon. Yields and selectivity were determined by gas chromatography. [a] propargylamine (1.5 mmol), DBU (0.15 mmol), solvent (1 mL), 25 °C and CO_2_ balloon. **c** The cyclization of various propargylamine with CO_2_.

A low-nuclear alkyne-protected Ag_4_ NC and the corresponding Ag_6_ NC and Ag_9_ NC were synthesized according to the literatures^45–47^. All these Ag NCs were characterized by mass spectrometry, UV‒vis absorption spectroscopy, and single-crystal diffraction analysis (Fig. 1a and Supplementary Figs. S1–S3), confirming the atomic monodispersity and the exact formula assigned to Ag_4_ NC, Ag_6_ NCand Ag_9_ NC, respectively. N-Benzylprop-2-yn-1-amine (**1a**, HC≡CCH_2_NHBn) was selected as the preferred substrate for the cyclization of propargylamine to explore the catalytic performance of the customized Ag_4_ NC. As expected, the Ag_4_ NC protected by the acetylene ligand showed the best performance. To exclude the influence of the number of metal atoms, we designed and synthesized Ag_2_ NC through a controlled experiment and compared their activity (Fig. 1a and Supplementary Fig. 4). Interestingly, among the Ag_n_ (*n* = 2,4,6,9) NC series, Ag_4_ NC had the highest catalytic activity with TON and TOF values up to 5746.2 and 2873.1 h^−1^, respectively, which were higher than those of reported catalysts (Fig. 1b and Supplementary Table 3). Then, we investigated the catalytic activity of AgNO_3_, AgBF_4_ and [Ag(C≡C^t^Bu)]_n_, and the results show that the activity of these catalysts is low. Furthermore, the Ag_4_ NC with a Dppf (1,1’-Bis(diphenylphosphino)ferrocene) ligand was inactive for this reaction (Fig. 1b and Supplementary Table 1). The changes in the kinetics of the cyclization of N-benzylprop-2-yn-1-amine with CO_2_ catalyzed by low-nuclear Ag_4_ NC were monitored by in situ ^1^H NMR (Supplementary Fig. 5a). Under the ideal conditions, we further explored the generality of the reaction for various propargylamine substrates. As shown in Fig. 1c and Supplementary Table 2, Ag_4_ NC afforded the target products in high yields within 2 h for all propargylamine substrates (**3a-4a**) with alkyl terminations. Moreover, Ag_4_ NC also reacted satisfactorily and afforded the corresponding products for substrates (**5a-7a**) with either electron-withdrawing or electron-donating groups. Most studies have reported that the low nucleophilicity of substrates such as N-phenylpropyl-2-yn-1-amine (**2a**) prevents the nucleophilic attack of carbon dioxide due to the benzene ring, resulting in a carbamate intermediate that is difficult to convert smoothly or requires high temperature conditions^16,48^. Much to our surprise and delight, the customized Ag_4_ NC achieved highly active conversion of the N-phenylpropyl-2-yn-1-amine substrate at room temperature with yields up to 87%.

**Fig. 2:**
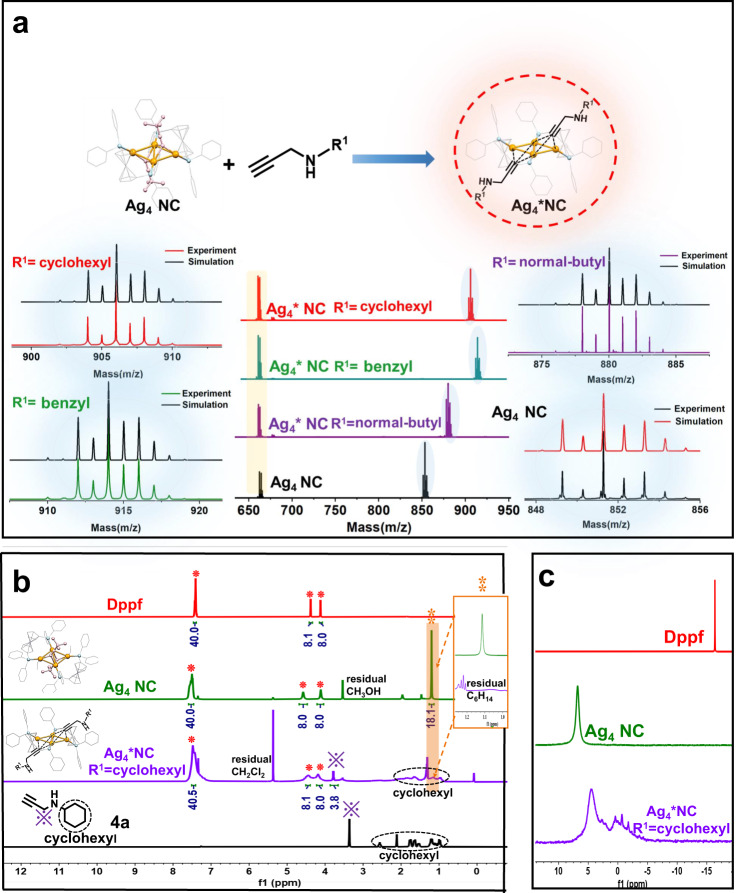
Characterization of Ag_4_ NC and Ag_4_* NC. **a** ESI-MS spectra of the intermediate Ag_4_* NC and simulation of the corresponding mass spectrum. **b** ^1^H NMR spectra of **4a**, Ag_4_* NC R^1^ = cyclohexyl, Ag_4_ NC, and Dppf. ∗ in red (Characteristic hydrogen of Dppf) ※ in purple (Characteristic hydrogen of the methylene group of N-2-Propyn-1-ylcyclohexanamine) ⁑ in orange (The methyl hydrogen peak (1.11 ppm) of the C≡C^t^Bu ligand disappears.) **c** ^31^P NMR spectra of Ag_4_* NC R^1^ = cyclohexyl, Ag_4_ NC, and Dppf.

On this basis, we conducted relevant control experiments to gain more insight into the fundamental source of the catalytic activity of Ag_4_ NC. The characteristic UV peak of Ag_4_ NC showed a slight blueshift (8 nm) after Ag_4_ NC was mixed with substrate 1a (1:2) for 1 h. In contrast, the characteristic peak of Ag_4_ NC did not change at all within 2 h (Supplementary Fig. 5a). The adsorption of **1a** on the Ag_4_ NC was detected by Fourier Transform Infrared (FT-IR) Spectroscopy. As shown in Supplementary Fig. 5c, the dominant stretching peak of C≡C-H at 3290 cm^−1^ disappeared, and the peak of C≡C at 2106 cm^−1^ shifted to 2120 cm^−1^. This reveals that the H atom of C≡C-H was removed from **1a** and that the C≡C bond of **1a** was activated by Ag_4_ NC, which was related to the dehydrogenation activation of **1a**^49^. To obtain direct evidence of the interaction between Ag_4_ NC and **1a**, we captured the Ag_4_* NC intermediate by ESI-MS. As shown in Fig. 2a, the mass spectrum peaks of Ag_4_* NC (R^1^ = benzyl) were detected and calculated to be 661.0 m/z and 914.0 m/z (Simulation: 661.3 m/z = [Ag+Dppf]^+^, 914.3 m/z = [1/2Ag_4_-C≡C^t^Bu + C≡CCH_2_NHBn-CH_3_OH]^+^ respectively). The ESI-MS peaks of Ag_4_* NC (R^1^ = benzyl) corresponded exactly to those of Ag_4_ NC (661.3 m/z and 851.3 m/z, Simulation: 661.3 m/z = [Ag+Dppf]^+^, 851.3 m/z = [1/2Ag_4_-CH_3_OH]^+^, respectively). Notably, Ag_4_* (R^1 ^= benzyl) species were also successfully detected by ESI-MS in the reaction solution (Ag_4_ + **1a** + CO_2_), suggesting that Ag_4_* NC is a key intermediate in the catalytic cycle (Supplementary Fig. 6a). To confirm this hypothesis, we isolated and verified the activity of Ag_4_* NC (R^1 ^= benzyl). The experimental results showed that the activity of Ag_4_* NC and Ag_4_ NC was similar, confirming that Ag_4_* NC was the key intermediate. The isolated Ag_4_* NC (R^1^=cyclohexyl), Ag_4_ NC, dppf ligand and substrate **4a** were characterized by ^1^H NMR (Fig. 2b). The ^1^H NMR spectrum of Ag_4_ NC contains the characteristic peak attributed to the hydrogen of the dppf and tert-butylvinyl ligands, and the ratio of the intensities of the peaks attributed to the benzene ring hydrogen (7.49 ppm) on the dppf ligand to the metal ring hydrogen (4.49 ppm, 4.03 ppm) and the C≡C^t^Bu ligand methyl hydrogen (1.11 ppm) was 40:8:8:18, and some peak shifts were observed. This was consistent with the molecular formula of Ag_4_ NC, which reflects the structural integrity and high matching of the Ag_4_ NC. Compared with the ^1^H NMR spectrum of **4a**, the ^1^H NMR spectrum of Ag_4_* NC showed shifts in the characteristic peak of the hydrogen of the cyclohexyl group (marked by the black dashed circle) and the methylene hydrogen peak (purple symbol) in the substrate HC≡CCH_2_NHCy (**4a**), while the methyl hydrogen peak of the C≡C^t^Bu ligand (1.11 ppm) disappeared. Additionally, the ratio of the intensities of the peaks attributed to the methylene hydrogen of C≡CCH_2_NHCy (3.69 ppm), the monocyclic hydrogen of the dppf ligand (4.10 ppm, 4.34 ppm) and the benzene ring hydrogen (7.40 ppm) was 3.8:8:8:40.5, indicating that the structure of the Ag_4_* NC molecule was similar to that of the Ag_4_ NC molecule, including two dppf ligands and two C≡CCH_2_NHCy ligands. At the same time, it can be seen from the ^31^P spectrum (Fig. 2c) that the structure of Ag_4_* NC is similar to that of Ag_4_ NC, and there is no free P ligand in the system. Moreover, the other substrates [HC≡CCH_2_NHCy (**4a**, R^1 ^= cyclohexyl) and HC≡CCH_2_NH^n^Bu (**3a**, R^1 ^= normal-buty)] were selected for the primitive reaction with Ag_4_ NC. The ESI-MS results showed two ionic peaks located at 661.0 m/z and 906.0 m/z (Simulation: 661.3 m/z = [Ag+Dppf]^+^, 906.3 m/z = [1/2Ag_4_-C≡C^t^Bu+C ≡ CCH_2_NHCy-CH_3_OH]^+^, respectively), along with peaks at 661.0 m/z and 880.0 m/z (Simulation: 661.3 m/z = [Ag+Dppf]^+^, 880.3 m/z = [1/2Ag_4_-C≡C^t^Bu + C≡CCH_2_NH^n^Bu-CH_3_OH]^+^, respectively). Meanwhile, the Ag_4_* (R^1 ^= cyclohexyl) species was also successfully identified in the reaction solution (Ag_4_ + **4a** + CO_2_) (Fig. 2a and Supplementary Fig. 6b).

**Fig. 3:**
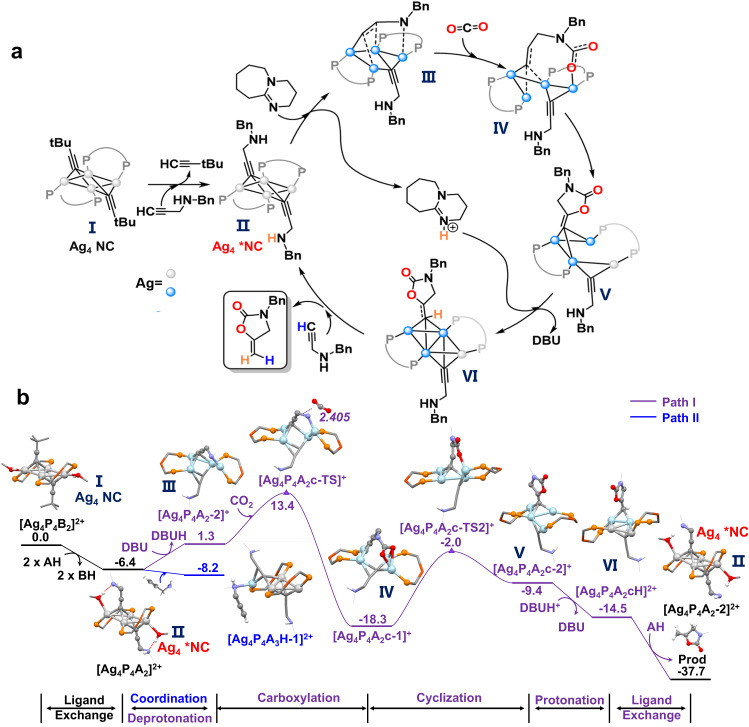
Proposed mechanism and calculation of the relative Gibbs free energies for Ag_4_ NC. **a** Proposed mechanism by Ag_4_ NC. **b** The relative Gibbs free energies, in bold. Gibbs free energy profiles of the Ag_4_ NC on carboxylation of N-benzylprop-2-yn-1-amine. Abbreviated labels: AH (N-benzylprop-2-yn-1-amine,**1a**); BH (3,3-Dimethyl-1-butyne); c(CO_2_); P_2_(dppf). For clarity, the two MeOH molecules, all H atoms (unless the reaction site), and the benzyl group on N-benzylprop-2-yn-1-amine were omitted in all structures except for Ag_4_P_4_B_2_ and Ag_4_P_4_A_2_. Silver: silver and light blue.

Consistent with the experimental observations, the ligand exchange of 3,3-dimethyl-1-butyne (BH) with N-benzylprop-2-yn-1-amine (AH) was found to be thermodynamically feasible (exergonic by 6.4 kcal/mol, Fig. 3b). After that, two main types of mechanisms, depending on whether carboxylation occurs on the incoming A substrate (via ligand exchange, Path I) or an extra AH substrate (Path II, Supplementary Fig. 15 and Fig. 3b), were investigated. In path I, the coordinated A group on Ag_4_P_4_A_2_ first reacted with DBU, and this step was slightly endergonic by 7.7 kcal/mol (Fig. 3b). Thereafter, carboxylation with CO_2_ occurred on Ag_4_P_4_A_2_-2 to generate the intermediate Ag_4_P_4_A_2_c-1 (c represents CO_2_), with a low activation barrier of 12.1 kcal/mol owing to the high nucleophilicity of the deprotonated amino group. Subsequent cyclization then occurred with a barrier of 16.3 kcal/mol. The resulting intermediate Ag_4_P_4_A_2_c-2 then underwent protonation and ligand exchange to complete the catalytic cycle. Overall, the Ag_4_-catalyzed cycloaddition of N-benzylprop-2-yn-1-amine was highly exergonic by -37.7 kcal/mol, and the carboxylation step was the rate-determining step (Ag_4_P_4_A_2_-2 → Ag_4_P_4_A_2_c-1). Path II started with the coordination of an extra AH substrate, preferentially via an amino group, to form Ag_4_P_4_A_3_H-1 (Supplementary Figs. 15, 16 and Fig. 3b). Similar to the overall transformation in Path I, deprotonation, carboxylation, cyclization, and protonation then occurred to generate the final product. However, the overall energy demands for Path II were 4.4 kcal/mol higher than those for Path I (26.0 vs. 21.6 kcal/mol in Fig. 3b and Supplementary Fig. 15). Of note, in this study, some other pathways, including deprotonation and carboxylation on Ag_4_P_4_A_3_H-1, were also examined but were excluded because of their relatively high energy demands (Supplementary Fig. 17). In this context, Path I was the most feasible pathway. Moreover, the carboxylation process of path I was experimentally investigated by ^13^C NMR and ESI-MS. As shown in Supplementary Fig. 19, the ^13^C NMR carbon spectrum shows that the characteristic peaks of raw material **1a** gradually weakened with the insertion of carbon dioxide. Meanwhile, new peaks assigned to the products gradually emerge and enhance. The characteristic peak signal changed significantly within 0.5 h, so we monitor the ESI-MS spectrum of the reaction solution during this period. To be noted, intermediate species IV (Fig. 3) was successfully detected by ESI-MS when Ag_4_ NC, **1a** and CO_2_ were mixed for 15 min. The mass peak of [Ag_4_C≡CCH_2_NHBnC=CCH_2_CH_2_O_2_NBn (Dppf)_2_]^+^ was detected at 1871.6 m/z (simulation: 1871.6 m/z) (Supplementary Fig. 20), coincident with the species IV on path I of DFT calculations (Fig. 3b, via ligand exchange). The tetranuclear Ag_4_ core was pivotal in stabilizing the deprotonated amino group in Ag_4_P_4_A_2_-2 and the anionic carboxylic group in Ag_4_P_4_A_2_c-1. Such an interaction was unlikely in the Ag_2_ system, as Ag-N coordination resulted in remarkable structural distortion in the diphosphine ligand. This was also the reason the yield of the Ag_2_ system was significantly lower than that of the Ag_4_ system (Supplementary Fig. 18). Based on the above, we proposed a mechanism for the cyclization of propargylamine with CO_2_ catalyzed by Ag_4_ NC (Fig. 3a). Obviously, Ag_4_ NC first interacted with the propylamine substrate to produce the dehydrogenation activation product Ag_4_* NC, which remained in the form of Ag_4_* NC after cyclization. Throughout the catalytic process, activation of the substrate required coordination between multiple Ag atoms (the blue atoms represent the active Ag atoms), confirming the reaction pathways that are inaccessible to the Ag single-atom catalyst and Ag_2_ NC.

**Fig. 4:**
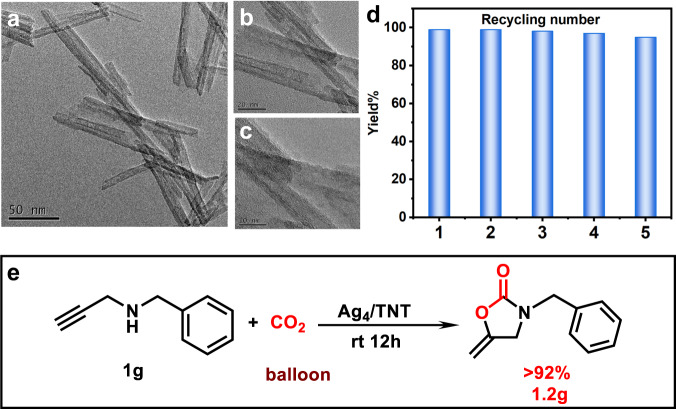
Characterization and applications of Ag_4_/TNT. **a**–**c** TEM images of Ag_4_/TNT. **d** Recoverability of Ag_4_/TNT catalysts in the cyclization of propargylamine with CO_2_. reaction conditions: Ag_4_/TNT (50 mg, 1.6 wt% loading of NCs), N-benzylprop-2-yn-1-amine (0.5 mmol), DBU (0.05 mmol), acetonitrile (1.0 mL), 25 °C, 12 h, CO_2_ balloon. **e** Gram scale experiment.

To understand its applicability, the Ag_4_/TNT nanocomposite was successfully synthesized, which characterized by solid-state UV, XRD, TEM, XPS and element mapping (see Fig. 4 and Supplementary Figs. 7–11 for details). The Ag_4_/TNT nanocomposites demonstrated the same activity as Ag_4_ NC, while TNT carrier was inactive (Supplementary Fig. 12). In this scenario, a recycling experiment was performed with **1a** as the substrate, and the reaction efficiency did not show significant changes even after five runs (Fig. 4d and Supplementary Fig. 13). To determine the practicability of this transformation, a scale-up experiment afforded 3-benzyl-5-methylene-2-oxazolone in 1.2 g and >92% yield, which is comparable to previous results (Fig. 4e).

In summary, alkyne-protected low-nuclear Ag_4_ nanocluster (NC) is designed to catalyze the cyclization of propargylamine with CO_2_. As expected, the low-nuclear Ag_4_ NC achieves the highest TON value of 5746.2, significantly higher than that of reported catalysts and the corresponding Ag_2_ NC, Ag_6_ NC and Ag_9_ NC. In addition, the Ag_4_ NC successfully achieves the cyclization of propargylamine with CO_2_ under mild conditions. In the elementary reaction of Ag_4_ NC with substrates, including HC≡CCH_2_NHBn, HC≡CCH_2_NHCy and HC≡CCH_2_NH^n^Bu, we capture three Ag_4_* NC intermediates and confirm their structures by Electrospray ionization (ESI). Density functional theory (DFT) calculations further confirm the key role of four Ag atoms, revealing the feasibility and importance of low-nuclear catalysts at the atomic level. Importantly, the Ag_4_/TNT (functional titanate nanotubes) nanocomposite afford the product at the gram scale. Therefore, the customized Ag_4_ catalyst improves the reaction activity while exerting the atomic economy similar to that of single atom catalyst, which has advantages in reducing cost. The present work provides a new perspective on the mechanism of the cyclization of propargylamine with CO_2_, which provides further support for the design of further atomic level catalysts and their efficient utilization.

## Methods

### Characterizations

The UV−vis. spectra were recorded on a Techcomp UV 1000 spectrophotometer. Transmission electron microscopy (TEM) was conducted on a JEM-2100 microscope with an accelerating voltage of 200 kV. The FT-IR spectra were recorded with a Bruker Tensor 27 instrument. The X-ray diffraction (XRD) patterns were obtained on Smart Lab 9 KW with Cu Kα radiation. The NCs loaded on the TNT catalyst support were determined by Inductively Coupled Plasma Mass Spectrometry (ICP-MS). The X-ray photoelectron spectroscopy (XPS) measurements were conducted on ESCALAB 250Xi. Electrospray ionization mass spectra (ESI-MS) were recorded using a Waters UPLC H-class/Xevo G2-XS Qtof mass spectromete.

### Catalytic activity

A typical “the cyclization of propargylamine with CO_2_” reaction was used to evaluate the catalytic performance of Ag_4_ NC. Ag_4_ NC (0.4 mg, 0.2×10^−3^ mmol), propargylamines (0.5 mmol), and 1,8-Diazabicyclo [5.4.0] undec-7-ene(DBU) (0.05 mmol) were added to acetonitrile (1 mL) in the reaction tube. The reaction stirring for 2 h at 25 °C with the balloon in Carbon dioxide atmosphere. After the reaction stopped, The reaction solution was diluted by dichloromethane, The conversion and selectivity were determined by GC analysis and column chromatography (EtOAc/PE = 1:5).

### Supplementary information

Supporting Information

Peer Review File

## Data Availability

Data supporting the findings of this work are available within the article and its Supplementary Information. The data that support the findings of this study are available from the corresponding author upon request. The X-ray crystallographic structures reported in this work have been deposited at the Cambridge Crystallographic Data Center (CCDC) under deposition numbers 2254886 for [Ag_2_dppf_3_]. These data can be obtained free of charge from the CCDC via https://www.ccdc.cam.ac.uk/structures/.
