# Supplementary material for: A low-nuclear Ag4 nanocluster as a customized catalyst for the cyclization of propargylamine with CO2
Source: Nat Commun. 2023 Nov 1;14:6989. doi: 10.1038/s41467-023-42723-3 (PMC10620197; doi:10.1038/s41467-023-42723-3)
Supplement: Supplementary file 1 — Supporting Information [file 41467_2023_42723_MOESM1_ESM.pdf]

Supplementary Information

A low-nuclear Ag_4 nanocluster as a customized catalyst for the cyclization of propargylamine with CO_2

Lin Li[1, 2, 3], Ying Lv[1, 2, 3], Hongting Sheng[1, 2, 3]*, Yonglei Du[1, 2, 3], Haifeng Li[1, 2, 3], Yapei Yun[2, 3], Ziyi Zhang[1, 2, 3], Haizhu Yu[1, 2, 3]*, Manzhou Zhu[1, 2, 3, 4]*

[1] Department of Chemistry and Centre for Atomic Engineering of Advanced Materials, Anhui University. [2] Key Laboratory of Structure and Functional Regulation of Hybrid Materials of Ministry of Education, [3] Key Laboratory of Functional Inorganic Material Chemistry of Anhui Province, Anhui University, Hefei 230601, China. [4] Anhui Tongyuan Environment Energy Saving Co., Ltd, Hefei, Anhui Province, PR China.

Lin Li and Ying Lv contributed equally.

E-mail: zmz@ahu.edu.cn; shenght@ahu.edu.cn; yuhaizhu@ahu.edu.cn.

Table and Contents

Supplementary Methods S1-S8

Supplementary Figures S9-S31

Supplementary Tables S32-S35

Supplementary Reference: S35-S39

Supplementary Methods

Abbreviations:

NC: nanocluster

TNT: functional titanate nanotubes

Materials:

All reagents were commercially available and were used as received without further purification, including silver nitrate ($AgNO_3$, 99%), Silver acetate ($C_2H_3O_2Ag$, 99.5%), 1,2-benzenedithiol (1,2-BDT), 1,1'-Bis(diphenylphosphino)ferrocene (Dppf,99%), 3,3-Dimethyl-1-Butyne (C_6H_{10}, 96%), tetraoctylammoniumbromide (TOAB,98%), bis(diphenylphosphino)methane (Dppm,98%), Diphenylsilane ($(C_6H_5)_2SiH_2$,97%), 3-Phenyl-2-propyn-1-ol (C_9H_8O, 96%), Phosphorus tribromide (PBr_3, 1.0 M in methylene chloride), Pyridine (C_5H_5N, 99%), Propargyl bromide (C_3H_3Br, 80 wt% toluene solution containing 0.3 % MgO stabilizer), Fluoboric Acid (HBF_4, 50 wt. % in H_2O), phenylacetylene (C_8H_6, 97%), Benzylamine (C_7H_9N, 99%), 4-Fluorobenzylamine (C_7H_8FN, 99%), 4-Chlorobenzylamine (C_7H_8ClN, 99%), 4-Methoxybenzylamine ($C_8H_{11}NO$, 98%), n-Butylamine ($C_4H_{11}N$, 98%), Cyclohexylamine ($C_6H_{13}N$, 99%), Aniline ($C_6H_5NH_2$, 99%), sodium borohydride ($NaBH_4$, 99.99%), methanol (CH_3OH, HPLC grade, Aldrich), ethanol (CH_3CH_2OH, HPLC grade, Aldrich), n-hexane (Hex, HPLC grade, Aldrich), dichloromethane (CH_2Cl_2 , HPLC grade, Aldrich); the other chemicals were purchased from Aladdin (Shanghai, China). All glassware was thoroughly cleaned with aqua regia ($HCl:HNO_3$ = 3:1 v:v), copiously rinsed with pure water, and then dried in an oven prior to use.

Experimental Section:

1. Preparation of the $[(dppf)_2Ag_4(C{\equiv}C^tBu)_2(CH_3OH)_2]$ $(BF_4)_2{\cdot}2CH_3OH$ NC.

Synthesis of the $[AgC{\equiv}C^tBu]_n$: Ag_2O (12.9 mmol, 3 g) was slowly added to the round bottom flask containing 60ml Ammonium hydroxide (24 mmol, 840 mg), and the new silver ammonia solution was obtained under the condition of ice mixing, and then the clarified silver ammonia solution was carefully filtered. Under the condition of continuing stirring, 5 mL ethanol solution of 3,3-dimethyl-1-butyne (25.8 mmol, 2.12 g) was added to obtain

white precipitate. After the reaction for about 5-8 min, the reaction was filtered under reduced pressure, and then appropriate amounts of deionized water, absolute ethanol and absolute diethyl ether were added successively for washing and finally drying.

0.3 mmol $[AgC{\equiv}C^tBu]_n$ (0.057 g) was dissolved in 6 mL methanol solution of AgBF4 (0.058 g, 0.3 mmol) under ultrasonication in an ultrasonic bath at ambient temperature for 15 min. The ultrasonic frequency and the power (intensity) were 40 kHz and 150 W, respectively. Achieve rapid, high-quality, homogeneous solution is possible with the help of ultrasonic, which is very important in synthesis. To the resulting colorless solution, 0.2 mmol of dppf (0.11 g) was added which led to a change in the color of the solution from yellow to yellow-brown. Then the mixture was sealed and heated to 70℃ for 20 h. After cooling to ambient temperature, the solution was filtered. Slow evaporation of the obtained solution afforded the product the yellow crystals. Yield: 47.6% (9 mg, based on $[AgC{\equiv}C^tBu]_n$)

2. Preparation of the $[Ag_6H_4(dppm)_4(OAc)_2]$ NC.

To a stirring, white suspension of Ag(OAc) (100.0 mg, 0.599 mmol) in methylbenzene (1 mL) was added dropwise a solution containing 1,1‑ bis(diphenylphosphino)methane (233.3 mg, 0.606 mmol) and diphenylsilane (60.0 μL, 0.323 mmol) in methylbenzene (2 mL). This resulted in a rapid color change to dark red‑brown, concomitant with the deposition of an off‑white solid. After stirring at room temperature for 15 h, the reaction mixture was concentrated to 1.5 mL in vacuo. The mixture was then filtered through a medium porosity frit, and the collected solid was rinsed with diethyl ether (2 ~ 4 mL), methylbenzene (1 ~ 1.5 mL), tetrahydrofuran (1 ~ 0.5 mL), and again with diethyl ether (1 ~ 4 mL). The washings were then discarded. The obtained light gray powder is dissolved in dichloromethane and filtered. To the resulting paleyellow solution was added methylbenzene (1 mL). This solution was then layered with diethyl ether (8 mL). The solution was stored at -25℃ for 2 d, which resulted in the deposition of colorless crystals.

3. Preparation of the $[Ag_9(1,2\text{-}BDT)_6]$ $(TOA)_3$ NC.

This NC was prepared by dissolving $AgNO_3$ (20 mg, 0.117 mmol) in methanol. Then, a solution of the 1,2-BDT (13.5 μL, 0.117 mmol) ligand in 10 mL of DCM was added to

form a yellow turbid complex, indicating formation of the Ag−S bonds. The reaction mixture was reduced using a fresh aqueous solution of $NaBH_4$ (20 mg, 0.5 mmol) in the presence of TOAB (411.5 mg), resulting in a dark-brown solution that was left under continuous stirring for 90 min at room temperature. To purify the synthesized cluster, we centrifuged the solution at 6558 xg; the product consisted of a dark-brown precipitate that was neglected, and the dark-brown supernatant was dried under vacuum and washed several times with excess methanol to remove byproducts. The purified NC product (5 mg) was dissolved in DMF (2 mL) and filtered using a syringe filter. The cluster solution was layered with ethanol at 5℃.

4. Preparation of the $[Ag_2dppf_3]_n$ complex.

Synthesis of the $[Ag(C{\equiv}CCH_2NHCH_2\text{-}Ph)]_n$: Ag_2O (125 mg, 0.54 mmol) was slowly added to the round-bottom flask containing 6 mL Ammonium hydroxide (2.4 mmol, 84 mg), and the newly prepared silver ammonia solution was obtained under the condition of ice mixing. After carefully filtering the clarified silver ammonia solution, 1.5 mL deionized water was added, and stirring was continued for 5 min. N-benzylprop-2-yn-1-amine (152 μL, 1.025 mmol) was added to obtain white precipitate. After the reaction for about 20 min, the reaction was filtered under reduced pressure, and then appropriate amounts of deionized water, absolute ethanol and absolute ether were added successively for washing and finally drying.

0.3 mmol $[Ag(C{\equiv}CCH_2NHCH_2\text{-}Ph)]_n$ (0.075 g) was dissolved in 6 mL $AgBF_4$ (0.116 g, 0.6 mmol) methanol solution and ultrasonic suspension was obtained in room temperature ultrasonic bath. The solution was clarified by adding 2ml acetonitrile under ultrasonic condition, and then 0.2 mmol (0.11 g) of DPPF was added to change the color of the solution from yellow to orange-red. The mixture was then sealed, heated to 70℃ for 20 hours, cooled to ambient temperature and filtered. The resulting solution was slowly evaporated to produce orange-yellow crystals.

5.Peparation of Ag_4/TNT.

Synthesis of the TNT: For the synthesis of TNT via hydrothermal method, 1 g of TiO_2 powder was firstly added into 100 mL of 10 M NaOH solution. After 30 min of magnetic

stirring, the mixture was transferred into a 250 mL Teflon autoclave and heated at an 150 temperature for 24 hours. Afterwards, the suspension was cooled to room temperature and washed with distilled water for several times until the super-natant pH dropped to 7. A second acid washing was performed by adding hydrochloric acid HCl solution (1 M) drop-wise to super-natant in order to reach pH 3. After 3 h of acid washing under continuous magnetic stirring, the precipitate was again washed with distilled water several times until neutralization of superna-tant solution. Products were then dried at 70℃ overnight.

100 mg TNT was dissolved in 25 mL dichloromethane, then 2 mg metal Ag_4 NC were dissolved in 5 mL dichloromethane and poured into the above solution and mixed together. After stirring for 1 hours at room temperature, the solution was centrifuged at 7267 xg for 2 minutes, and the precipitate was washed with dichloromethane three times, then dried under vacuum.

6. Synthesis of substrates

To a solution of the corresponding primary amine (50 mmol, 5 eq.) in DCM (20 mL) was added dropwise a solution of 3-bromo-1-propyne in toluene (1.1 mL, 10 mmol) at room temperature. After stirring for 24 hours the reaction mixture was washed 3 times with 20 mL of water. The resulting organic phase was dried over Na_2SO_4, filtered and the solvent was evaporated under reduced pressure. The product was purified by column chromatography (pentane/EtOAc, 5:1)

NMR data of the products and NMR Spectra**.**

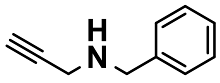

1a N-Benzyl-2-propynylamine

1H NMR (400 MHz, CHLOROFORM-D) δ 7.37 – 7.20 (m, 5H), 3.88 (s, 2H), 3.42 (d, J = 2.5 Hz, 2H), 2.26 (t, J = 2.4 Hz, 1H). 13C NMR (101 MHz, CHLOROFORM-D) δ 139.54, 128.55, 128.53, 127.27, 82.21, 71.68, 52.38, 37.44.

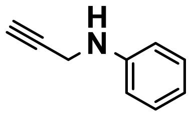

2a N-2-Propyn-1-ylbenzenamine

1H NMR (400 MHz, CHLOROFORM-D) δ 7.37 – 7.17 (m, 2H), 6.88 (t, J = 7.3, 1.2 Hz, 1H), 6.80 – 6.69 (m, 2H), 3.97 (d, J = 2.7 Hz, 2H), 2.30 (t, J = 2.2 Hz, 1H). 13C NMR (101 MHz, CHLOROFORM-D) δ 147.06, 129.44, 118.79, 113.72, 81.31, 71.51, 33.76.

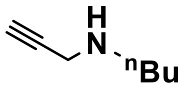

3a N-Butyl-2-propynylamine

^{1}H NMR (400 MHz, CHLOROFORM-D) δ 3.40 (d, J = 2.4 Hz, 2H), 2.67 (t, J = 7.1 Hz, 2H), 2.18 (t, J = 2.4 Hz, 1H), 1.51 – 1.39 (m, 2H), 1.40 – 1.28 (m, 2H), 0.90 (t, J = 7.3 Hz, 3H). 13C NMR (101 MHz, CHLOROFORM-D) δ 82.42, 71.23, 71.15, 48.45, 38.24, 32.00, 20.48, 14.02.

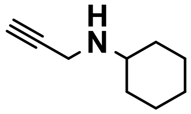

4a N-2-Propyn-1-ylcyclohexanamine

1H NMR (400 MHz, CHLOROFORM-D) δ 3.36 (t, J = 2.3 Hz, 2H), 2.62 – 2.51 (m, 1H), 2.11 (q, J = 2.2 Hz, 1H), 1.76 (d, J = 11.6 Hz, 2H), 1.70 – 1.58 (m, 2H), 1.58 – 1.49 (m, 1H), 1.29 – 0.90 (m, 6H). 13C NMR (101 MHz, CHLOROFORM-D) δ 82.55, 70.91, 55.16, 54.61, 35.05, 32.94, 26.06, 24.74.

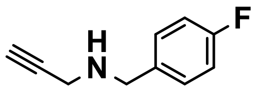

5a (4-Fluoro-benzyl)-prop-2-ynyl-amine

1H NMR (400 MHz, CHLOROFORM-D) δ 7.30 (dd, J = 8.4, 5.6 Hz, 2H), 6.99 (t, J = 8.7 Hz, 2H), 3.83 (s, 2H), 3.40 (d, J = 2.5 Hz, 2H), 2.25 (t, J = 2.4 Hz, 1H), 1.49 (s, 1H). 13C NMR (101 MHz, CHLOROFORM-D) δ 163.37, 160.93, 130.10, 130.02, 115.41, 115.20, 82.01, 71.73, 51.52, 37.29.

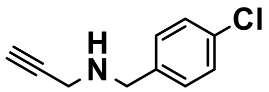

6a (4-Chloro-benzyl)-prop-2-ynyl-amine

1H NMR (400 MHz, CHLOROFORM-D) δ 7.28 (s, 4H), 3.84 (s, 2H), 3.40 (d, J = 2.4 Hz, 2H), 2.24 (t, J = 2.4 Hz, 1H), 1.48 (s, 1H). 13C NMR (101 MHz, CHLOROFORM-D) δ 137.97, 132.98, 129.83, 128.65, 81.93, 71.78, 51.54, 37.33.

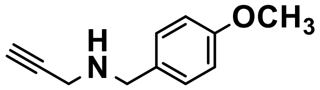

7a (4-Methoxy-benzyl)-prop-2-ynyl-amine

1H NMR (400 MHz, CHLOROFORM-D) δ 7.37 – 7.16 (m, 2H), 6.93 – 6.78 (m, 2H), 3.80 (s, 2H), 3.78 (s, 3H), 3.39 (d, J = 2.5 Hz, 2H), 2.24 (t, J = 2.4 Hz, 1H), 1.47 (s, 1H). 13C NMR (101 MHz, CHLOROFORM-D) δ 158.85, 131.56, 129.66, 113.87, 82.20, 71.52, 55.29, 51.69, 37.21.

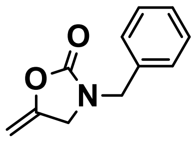

1b 3-Benzyl-5-methylene-2-oxazolidinone

1H NMR (400 MHz, CHLOROFORM-D) δ 7.50 – 7.11 (m, 5H), 4.71 (d, J = 2.9 Hz, 1H), 4.45 (s, 2H), 4.22 (d, J = 2.7 Hz, 1H), 4.00 (t, J = 2.4 Hz, 2H). 13C NMR (101 MHz, CHLOROFORM-D) δ 155.75, 149.06, 135.08, 129.07, 128.35, 128.27, 86.84, 47.94, 47.32.

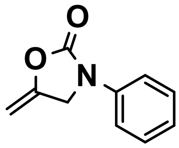

2b 5-Methylene-3-phenyl-2-oxazolidinone

1H NMR (400 MHz, CHLOROFORM-D) δ 7.53 (dd, J = 8.8, 1.2 Hz, 2H), 7.43 – 7.33 (m, 2H), 7.20 – 7.10 (m, 1H), 4.84 (q, J = 2.8 Hz, 1H), 4.61 (t, J = 2.4 Hz, 2H), 4.46 – 4.37 (m, 1H). 13C NMR (101 MHz, CHLOROFORM-D) δ 152.43, 147.81, 137.26, 129.31, 124.63, 118.18, 87.16, 48.47.

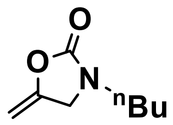

3b 3-Butyl-5-methylene-2-oxazolidinone

1H NMR (400 MHz, CHLOROFORM-D) δ 4.71 (d, J = 2.9 Hz, 1H), 4.31 – 4.23 (m, 1H), 4.13 (t, J = 2.4 Hz, 2H), 3.28 (t, J = 7.3 Hz, 2H), 1.51 (d, J = 7.6 Hz, 2H), 1.33 (d, J = 8.0 Hz, 2H), 0.92 (t, J = 7.3 Hz, 3H). 13C NMR (101 MHz, CHLOROFORM-D) δ 155.67, 149.25, 86.46, 47.85, 43.54, 29.35, 19.83, 13.69.

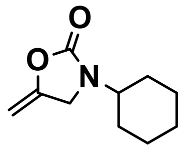

4b 3-Cyclohexyl-5-methylene-2-oxazolidinone

1H NMR (400 MHz, CHLOROFORM-D) δ 4.70 (q, J = 2.7 Hz, 1H), 4.25 (q, J = 2.4 Hz, 1H), 4.11 (t, J = 2.4 Hz, 2H), 3.79 – 3.61 (m, 1H), 1.80 (dt, J = 9.5, 2.6 Hz, 4H), 1.69 – 1.61 (m, 1H), 1.41 – 1.26 (m, 4H), 1.14 – 0.99 (m, 1H). 13C NMR (101 MHz, CHLOROFORM-D) δ 155.01, 149.74, 86.32, 52.45, 44.20, 30.36, 25.35, 25.32.

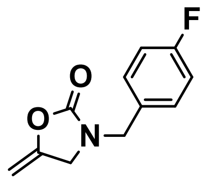

5b 3-[(4-Fluorophenyl)methyl]-5-methylene-2-oxazolidinone

1H NMR (400 MHz, CHLOROFORM-D) δ 7.39 – 7.18 (m, 2H), 7.11 – 6.96 (m, 2H), 4.73 (q, J = 2.7 Hz, 1H), 4.42 (s, 2H), 4.24 (dt, J = 3.2, 2.2 Hz, 1H), 4.00 (t, J = 2.4 Hz, 2H). 13C NMR (101 MHz, CHLOROFORM-D) δ 163.99, 161.53, 155.73, 148.91, 130.12, 130.04, 116.17, 115.96, 87.10, 47.28.

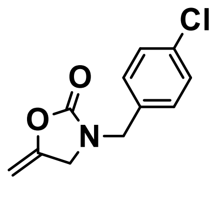

6b 3-[(4-Chlorophenyl)methyl]-5-methylene-2-oxazolidinone

^{1}H NMR (400 MHz, CHLOROFORM-D) δ 7.36 – 7.31 (m, 2H), 7.25 – 7.18 (m, 2H), 4.75 (d, J = 2.9 Hz, 1H), 4.43 (s, 2H), 4.31 – 4.19 (m, 1H), 4.01 (t, J = 2.4 Hz, 2H). 13C NMR (101 MHz, CHLOROFORM-D) δ 155.70, 148.78, 134.31, 133.61, 129.60, 129.27, 87.14, 47.28.

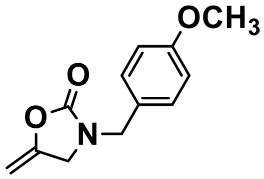

7b 3-[(4-Methoxyphenyl)methyl]-5-methylene-2-oxazolidinone

^{1}H NMR (400 MHz, CHLOROFORM-D) δ 7.18 (d, J = 8.7 Hz, 2H), 6.87 (d, J = 8.7 Hz, 2H), 4.70 (d, J = 2.9 Hz, 1H), 4.38 (s, 2H), 4.29 – 4.16 (m, 1H), 3.98 (t, J = 2.4 Hz, 2H), 3.79 (s, 3H). 13C NMR (101 MHz, CHLOROFORM-D) δ 159.64, 155.64, 149.12, 129.67, 127.06, 114.39, 86.72, 55.39, 47.33, 47.15.

7. Calculations of TON and TOF

To evaluate the efficiency of Ag_4 NC, the reaction of 1a and CO_2 was scaled up. propargylamine (1.5 mmol), 1,8-diazabicyclo[5.4.0]undec-7-ene (DBU, 0.05 mmol), and acetonitrile (1 ml) were mixed with Ag_4 (0.013 mol% of the substrate based on Ag_4 NC) and stirred at room temperature under a carbon dioxide atmosphere. After workup, 1a (1.12 mmol, 74.7% yield) was obtained. The TON was calculated by the equation (TON = Mole of products/mol of catalytic sites) and the value was given to be 5746.2. The TOF was calculated by the equation (TOF = Mole of products/mol of catalytic sites/time) and the value was given to be 2873.1h^{-1}.

8. Computational Details

All the density functional theory calculations were performed with the B3PW91 level of theory [1,2]on Gaussian 09 suite of program[3]. Silver atoms were treated with the SDD basis set and the related effective core potential (ECP), associating with f polarization function (ζ=1.611).[4] For the remaining atoms, the 6-31G(d,p) basis set was used. [5] Based on the optimized structures, the implicit solvent effects was taken into account via single-point calculations with SMD [6] model and the acetonitrile solvent (in accordance with the experiments), using the B3PW91 functional with a dispersion correction of GD3. [7,8] The similar theoretical method has been recently used for the transition metal catalyzed carboxylative cyclization of propargylamines reaction.[9]

Supplementary Figures

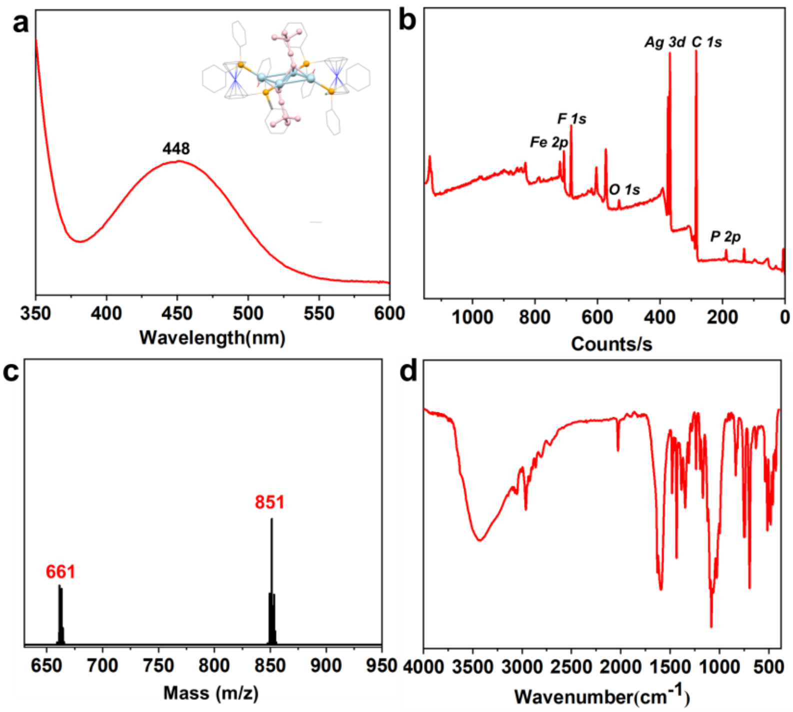

Supplementary Figure 1. (a) UV-vis. spectrum of Ag_4 NC (crystal structure diagram of Ag_4NC inner A, color label: pink, (-C≡C^{t}Bu); blue, Ag; dark, C; and orange, P; deep blue Fe). (b) XPS spectra of Ag_4 NC. (c) ESI-MS spectra of Ag_4 NC. (d) FT-IR spectra of Ag_4NC.

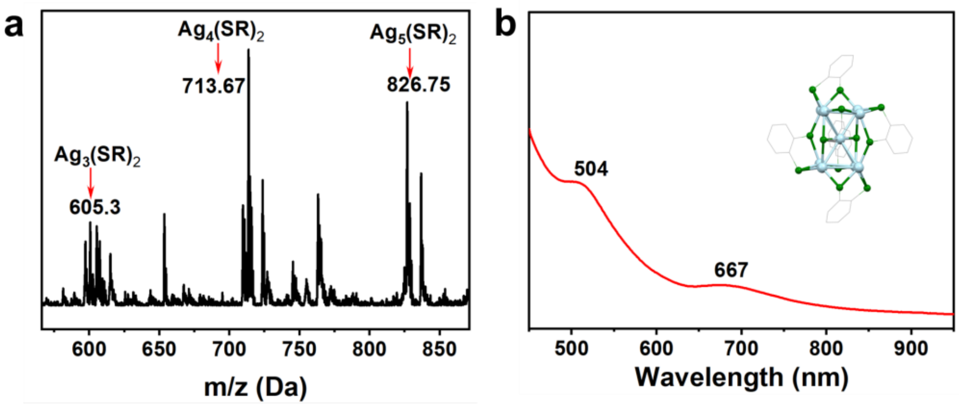

Supplementary Figure 2. (a) ESI-MS spectra of $[Ag_9(1,2\text{-BDT})_6]^{3-}$. (b) UV-vis. spectrum of $[Ag_9(1,2\text{-BDT})_6]^{3-}$ (crystal structure diagram of Ag_9 NC, color label: green,S; blue, Ag; dark, C).

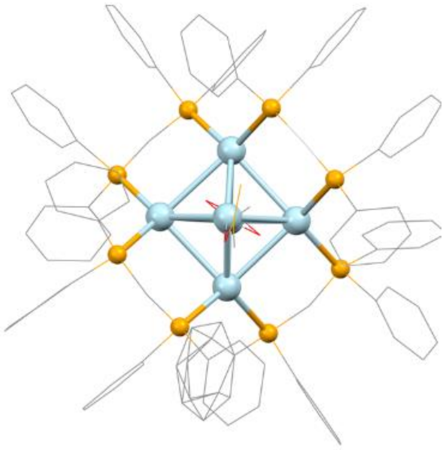

Supplementary Figure 3. Crystal structure diagram of [$Ag_6H_4(dppm)_4(OAc)_2$], color label: red, O; blue, Ag; dark, C; and orange, P).

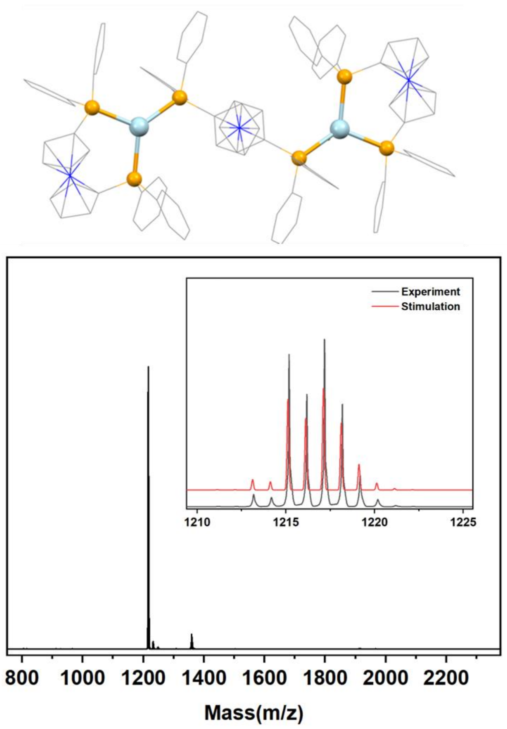

Supplementary Figure 4. ESI-MS spectra of $Ag_2(dppf)_3(BF_4)_2$. (crystal structure diagram of Ag_2 NC, color label: deep blue, Fe; blue, Ag; dark, C; and orange, P).

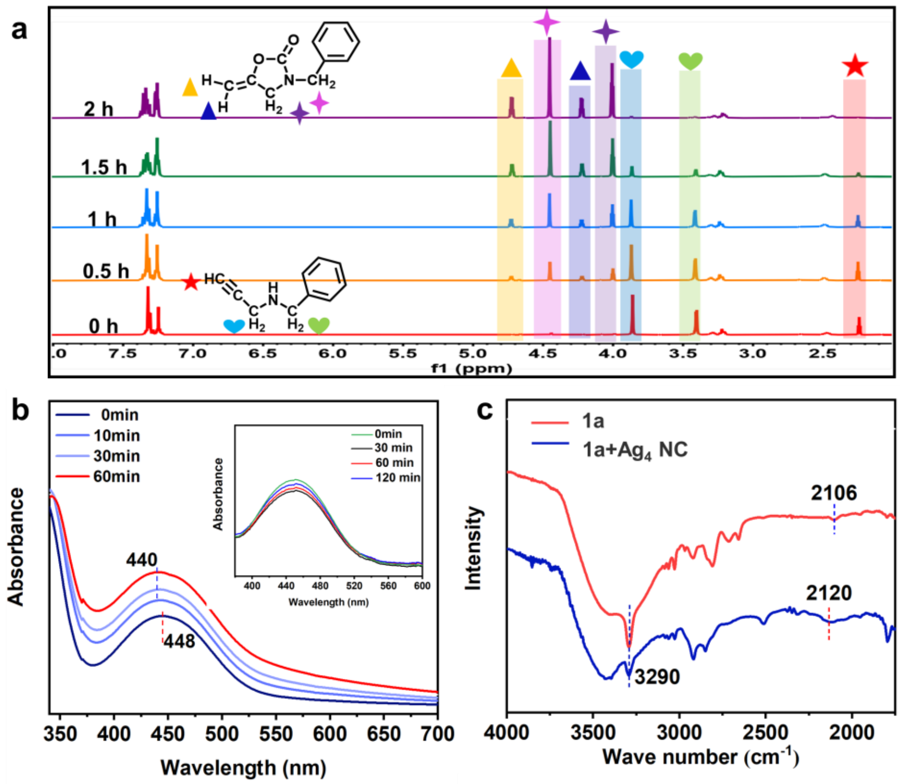

Supplementary Figure 5. (a) ^{1}H NMR spectra monitoring of CO_2 cycloaddition of N-benzylprop-2-yn-1-amine by Ag_4. (b)UV-vis. absorption spectrum of Ag_4NC mixed with 1a.(c) FT-IR spectra of 1a and the 1a+Ag_4 NC mixture.

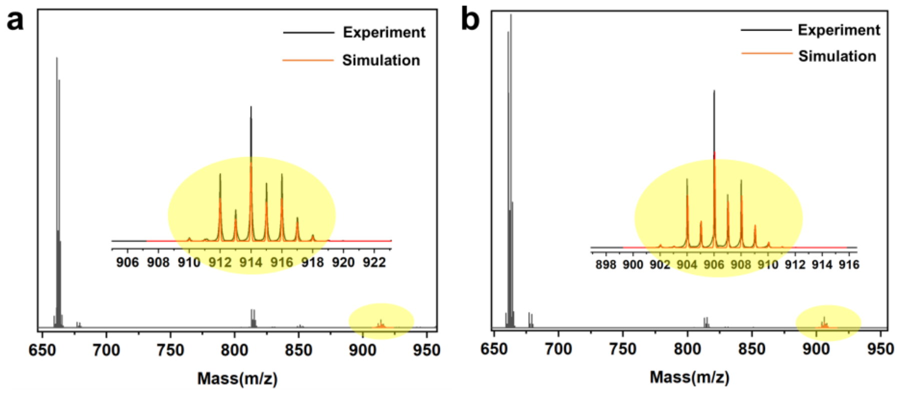

Supplementary Figure 6. (a) ESI-MS spectra of Ag_4NC mixed with 1a.(b) ESI-MS spectra of Ag_4NC mixed with 4a.

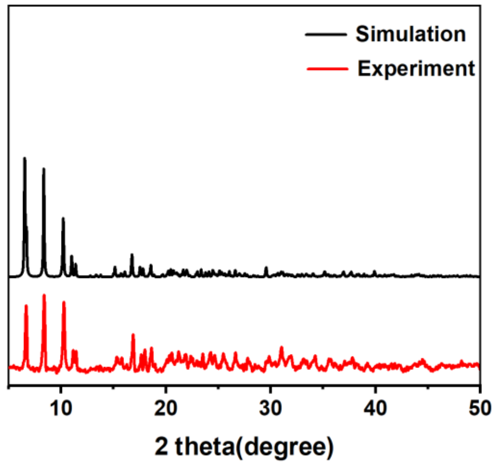

Supplementary Figure 7. XRD experimental data and simulation data of the Ag_4 NC.

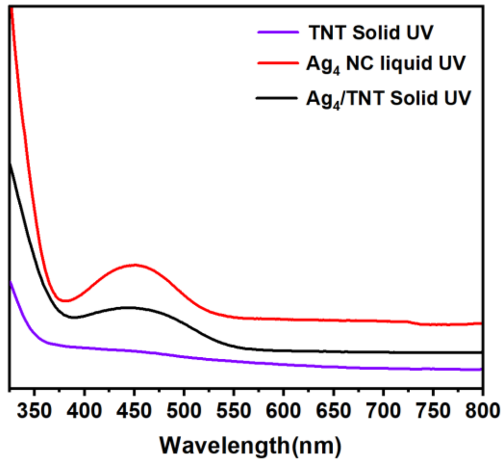

Supplementary Figure 8. Solid UV Absorption Spectroscopy of Ag_4/TNT、TNT and Liquid UV Absorption Spectroscopy of Ag_4 NC.

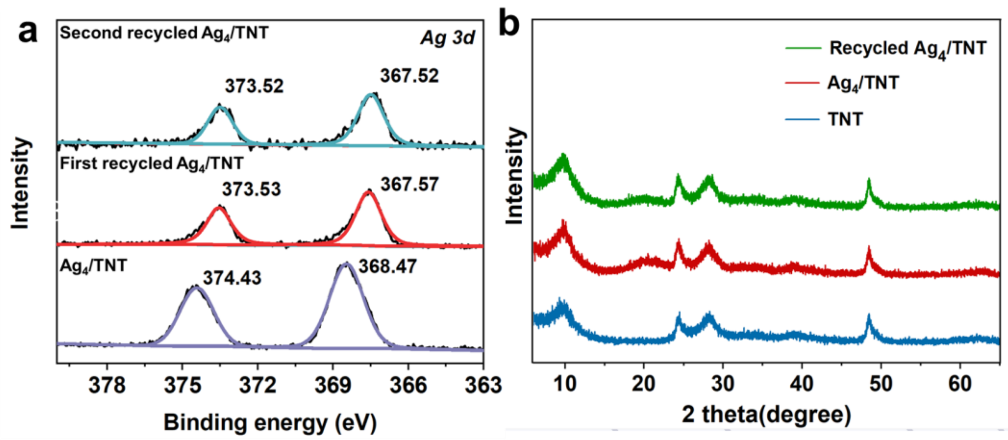

Supplementary Figure 9. (a). XPS spectrum of Ag_4/TNT and First recycled Ag_4/TNT and. Second recycled Ag_4/TNT (b). XRD patterns of TNT, Ag_4/TNT, and recycled Ag_4/TNT.

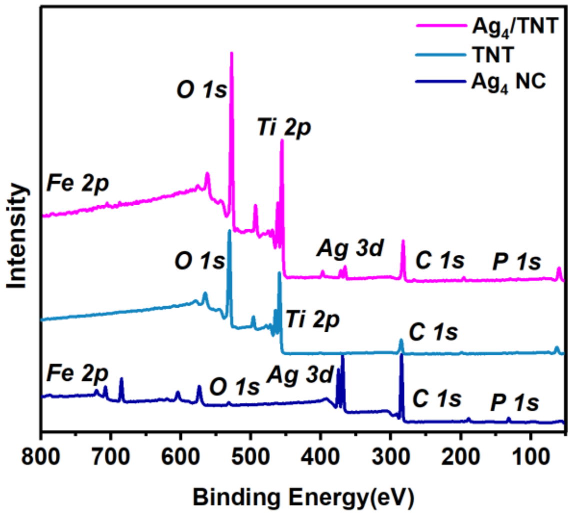

Supplementary Figure 10. XPS spectrum of Ag_4/TNT and Ag_4 NC and TNT.

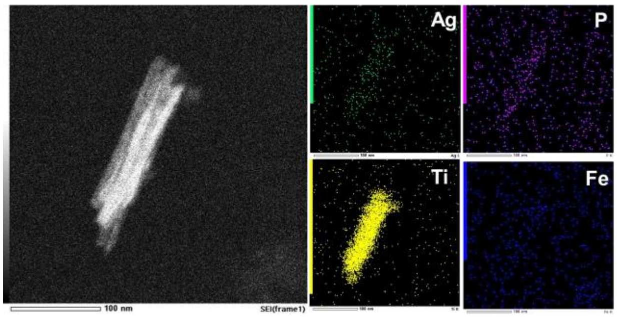

Supplementary Figure 11. TEM image and element maps for an Ag_4/TNT sample: Ag, Ti, Fe, P.

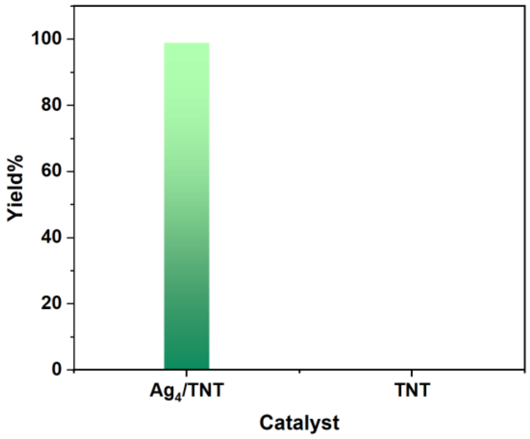

Supplementary Figure 12. Yield of Ag_4 NC catalyst for CO_2 cycloaddition of N-benzylprop-2-yn-1-amine. Reaction conditions: Ag_4/TNT (25 mg, 1.6wt% loading of NCs, 0.04mol% Ag_4 NC), N-benzylprop-2-yn-1-amine (0.5 mmol), DBU(0.05 mmol), acetonitrile (1.0 mL), 25 °C, reacting for 2 h with CO_2 ball.

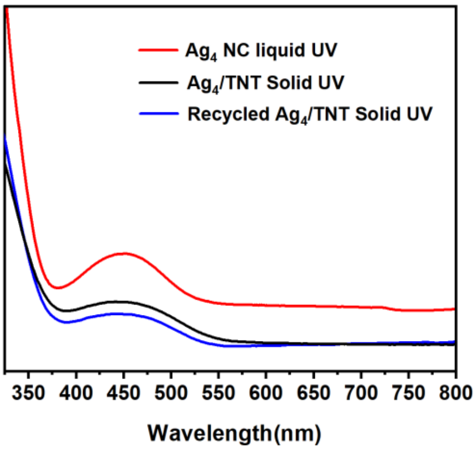

Supplementary Figure 13. Solid UV Absorption Spectroscopy of Ag_4/TNT and Recycled Ag_4/TNT and Liquid UV Absorption Spectroscopy of Ag_4 NC.

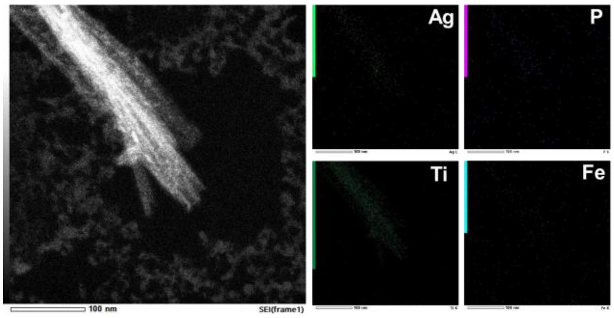

Supplementary Figure 14. TEM images and elemental maps of recycling Ag_4/TNT samples: Ag, Ti, Fe, P

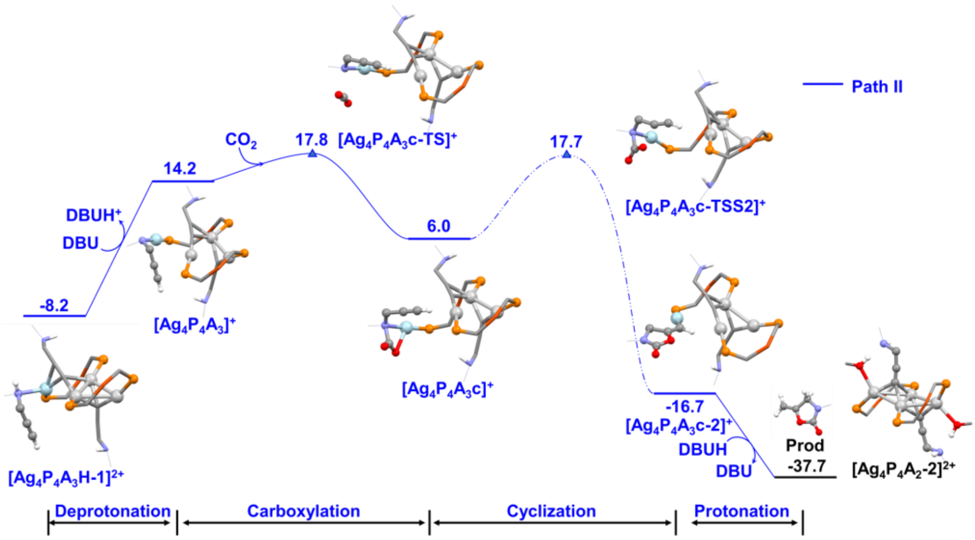

Supplementary Figure 15. The relative Gibbs free energies, in bold. Gibbs free energy profiles of the Ag_4 NC on carboxylation of N-benzylprop-2-yn-1-amine. (Path Ⅱ) Abbreviated labels: AH(N-benzylprop-2-yn-1-amine,1a); BH (3,3-Dimethyl-1-butyne); c (CO_2); P_2(dppf). For clarity, the two MeOH molecules, all H atoms (unless the reaction site), and the benzyl group on N-benzylprop-2-yn-1-amine were omitted in all structures except for $Ag_4P_4B_2$ and $Ag_4P_4A_2$. Silver: silver and light blue.

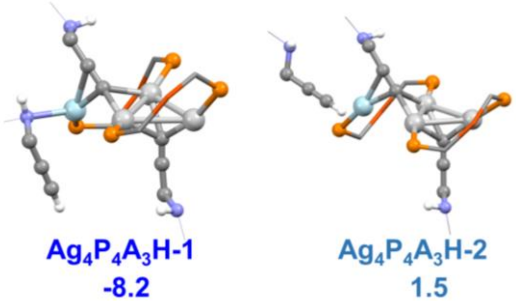

Supplementary Figure 16. The different coordination modes of the foreign ligands. The two MeOH were omitted. Silver: silver and light blue; N: blue; C: gray; H: white; O: red; P: orange.

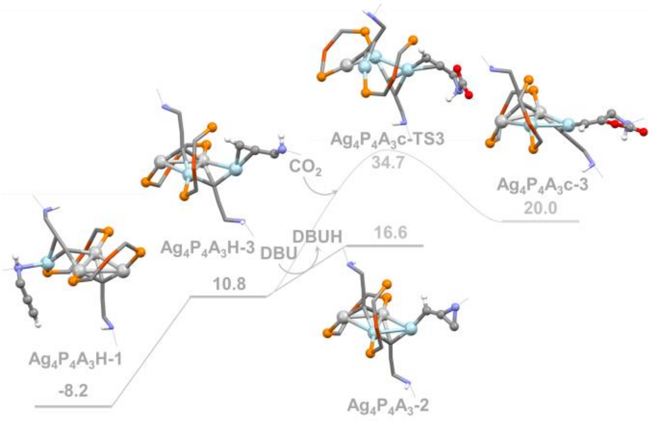

Supplementary Figure 17. The mechanism of Ag_4 catalyzed CO_2 cycloaddition of N-benzylprop-2-yn-1-amine from $Ag_4P_4A_3H$-1. The two MeOH molecules were omitted.

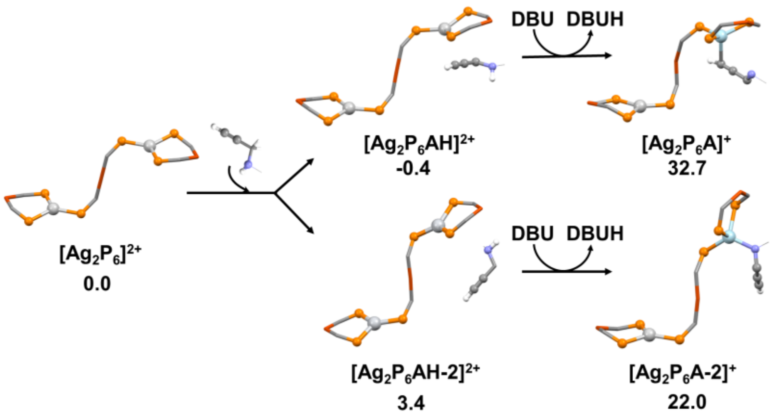

Supplementary Figure 18. The coordination of N-benzylprop-2-yn-1-amine on $[Ag_2P_6]^{2+}$.

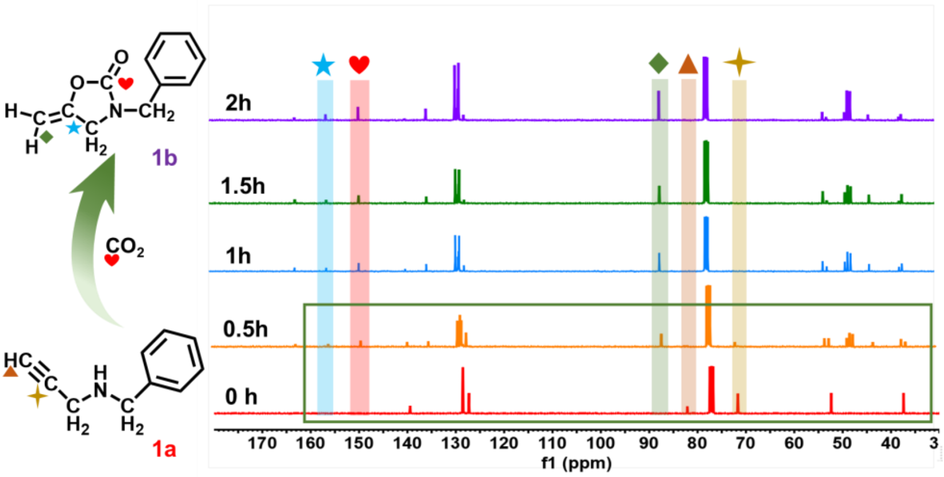

Supplementary Figure 19. ^{13}C NMR spectra monitoring of CO_2 cycloaddition of N-benzylprop-2-yn-1-amine by Ag_4 NC.

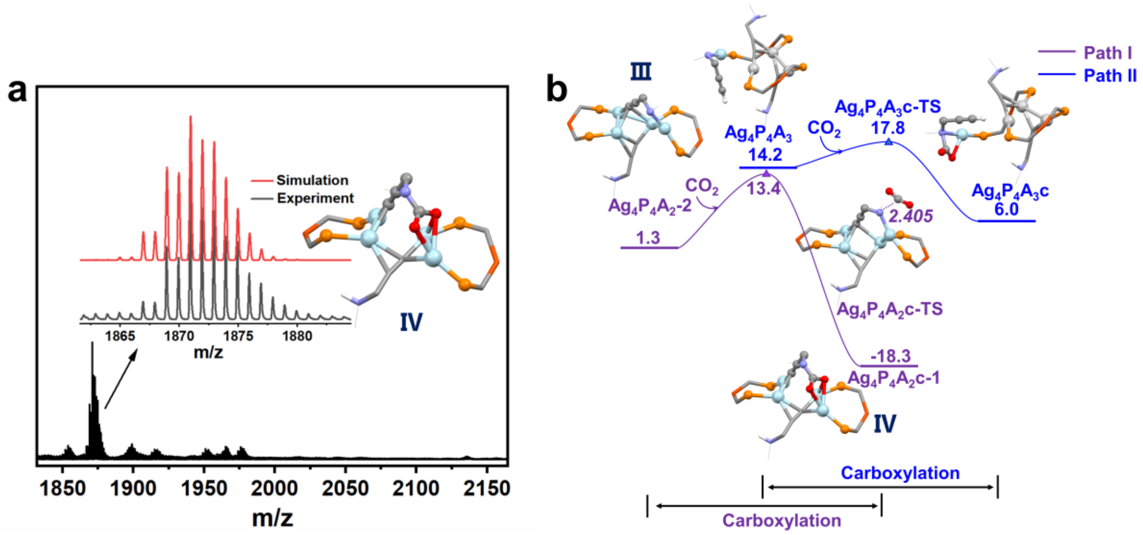

Supplementary Figure 20. (a) ESI-MS spectra of intermediates in the reaction process and simulation of the corresponding mass spectra. (b) the carboxylation process of path Ⅰ and path Ⅱ.

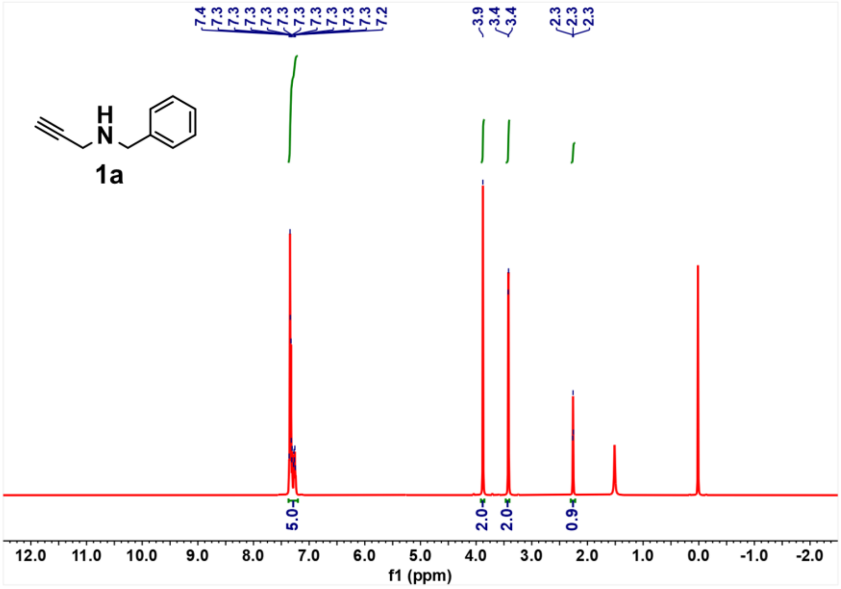

Supplementary Figure 21. ^{1}H NMR spectrum of N-Benzyl-2-propynylamine. (in $CDCl_3$)

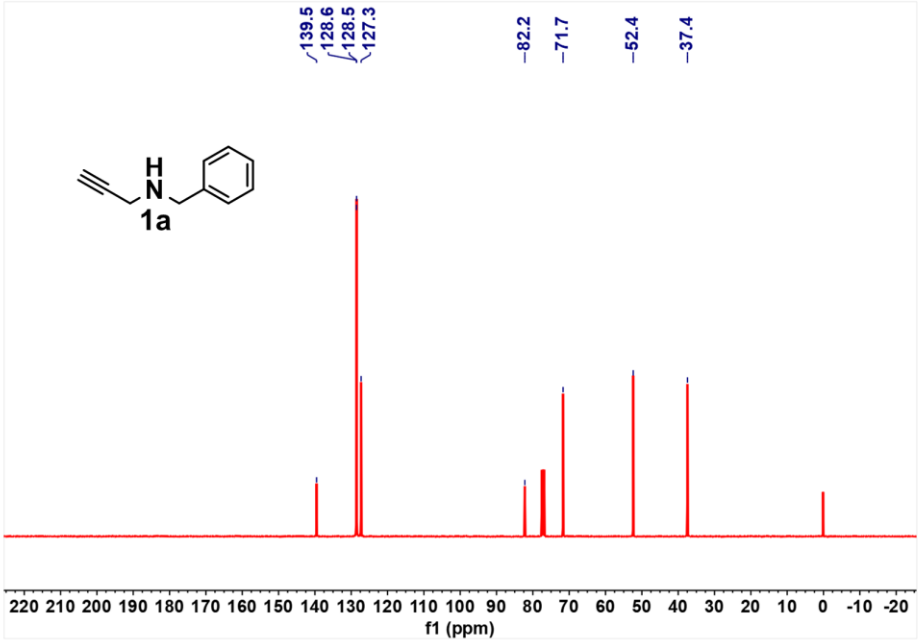

Supplementary Figure 22. ^{13}C NMR spectrum of N-Benzyl-2-propynylamine. (in $CDCl_3$)

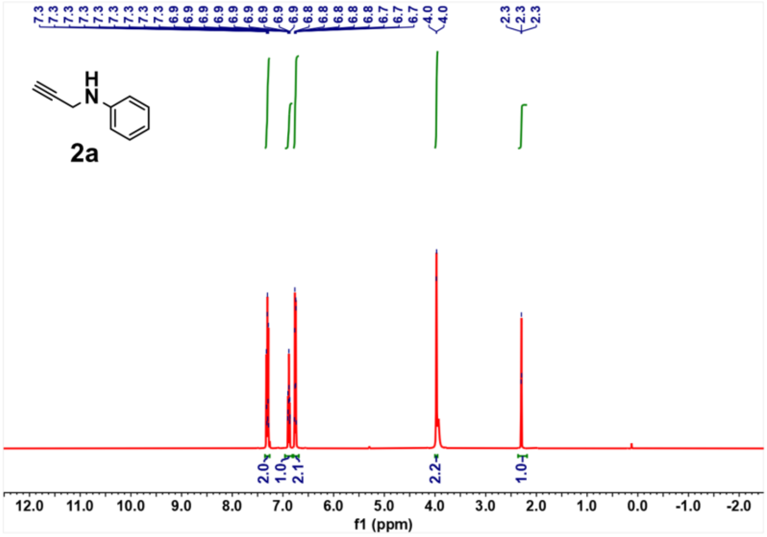

Supplementary Figure 23. ^{1}H NMR spectrum of N-2-Propyn-1-ylbenzenamine. (in $CDCl_3$)

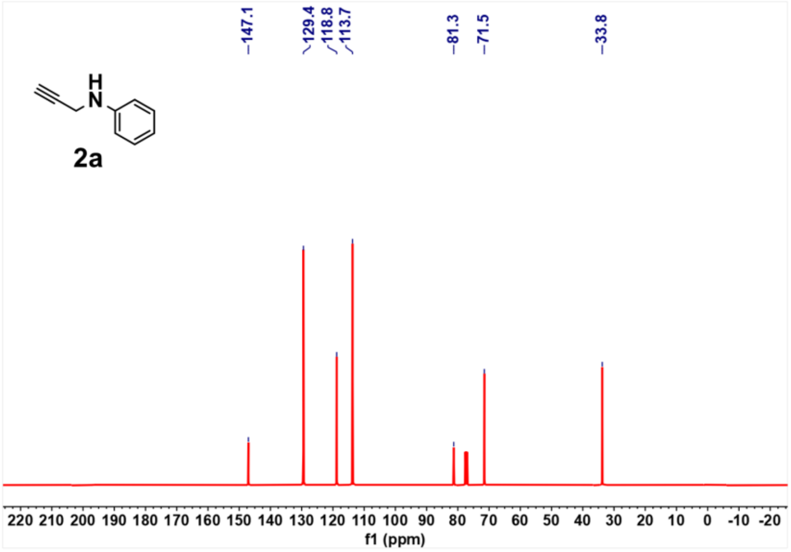

Supplementary Figure 24. ^{13}C NMR spectrum of N-2-Propyn-1-ylbenzenamine. (in $CDCl_3$)

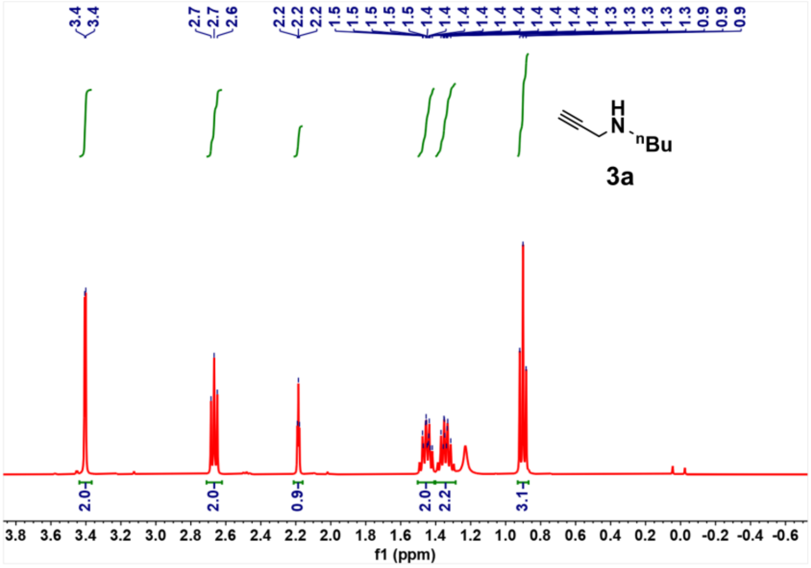

Supplementary Figure 25. ^{1}H NMR spectrum of N-Butyl-2-propynylamine. (in $CDCl_3$)

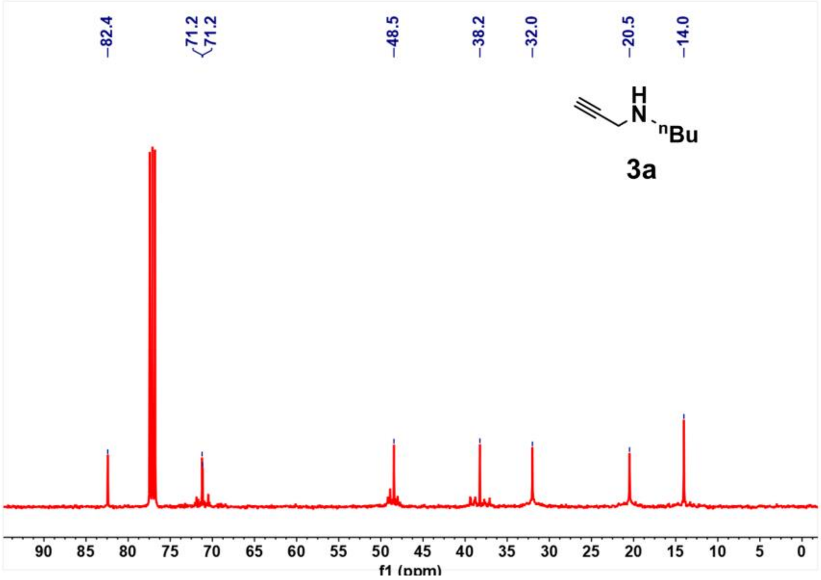

Supplementary Figure 26. ^{13}C NMR spectrum of N-Butyl-2-propynylamine. (in $CDCl_3$)

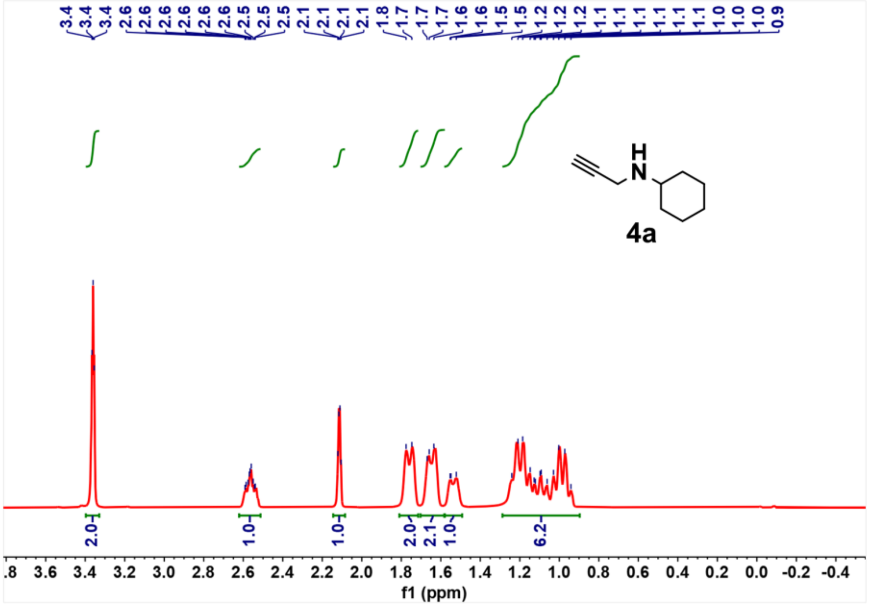

Supplementary Figure 27. ^{1}H NMR spectrum of 3-Cyclohexyl-5-methylene-2-oxazolidinone. (in $CDCl_3$)

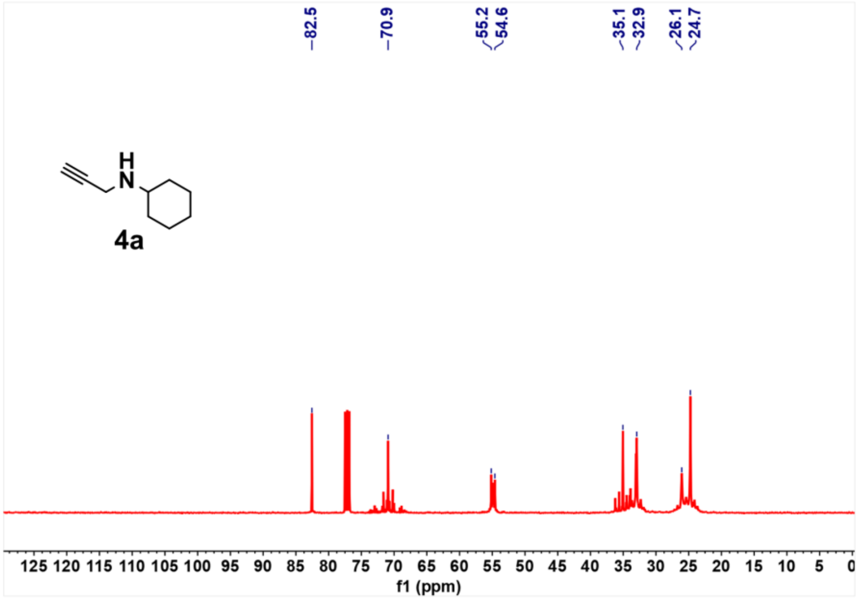

Supplementary Figure 28. ^{13}C NMR spectrum of 3-Cyclohexyl-5-methylene-2-oxazolidinone. (in $CDCl_3$)

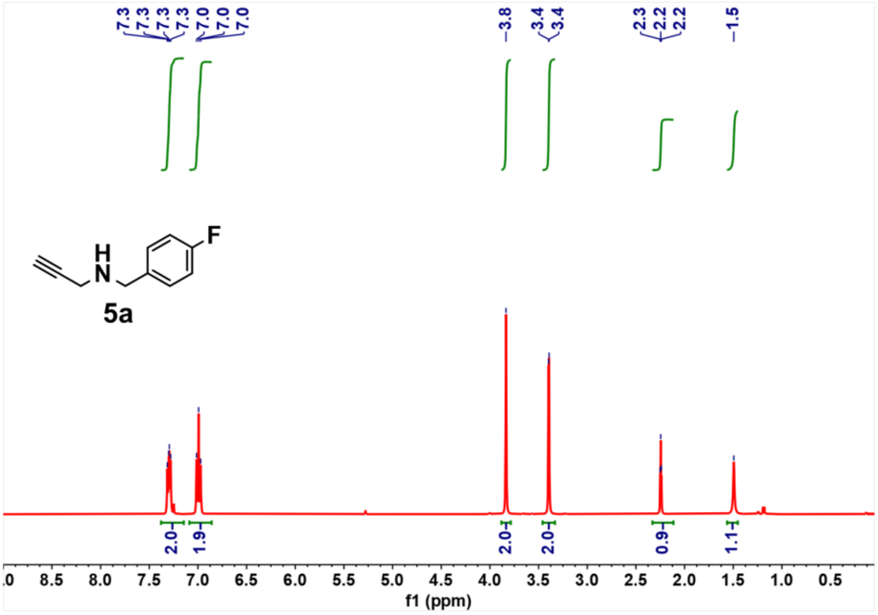

Supplementary Figure 29. ^{1}H NMR spectrum of (3-[(4-Fluorophenyl)methyl]-5-methylene-2-oxazolidinone. (in $CDCl_3$)

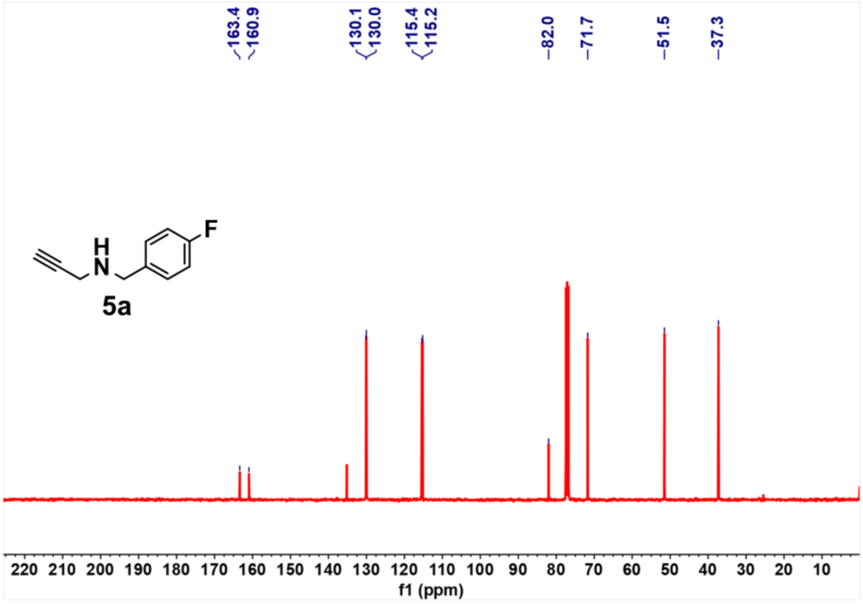

Supplementary Figure 30. ^{13}C NMR spectrum of 3-[(4-Fluorophenyl)methyl]-5-methylene-2-oxazolidinone. (in $CDCl_3$)

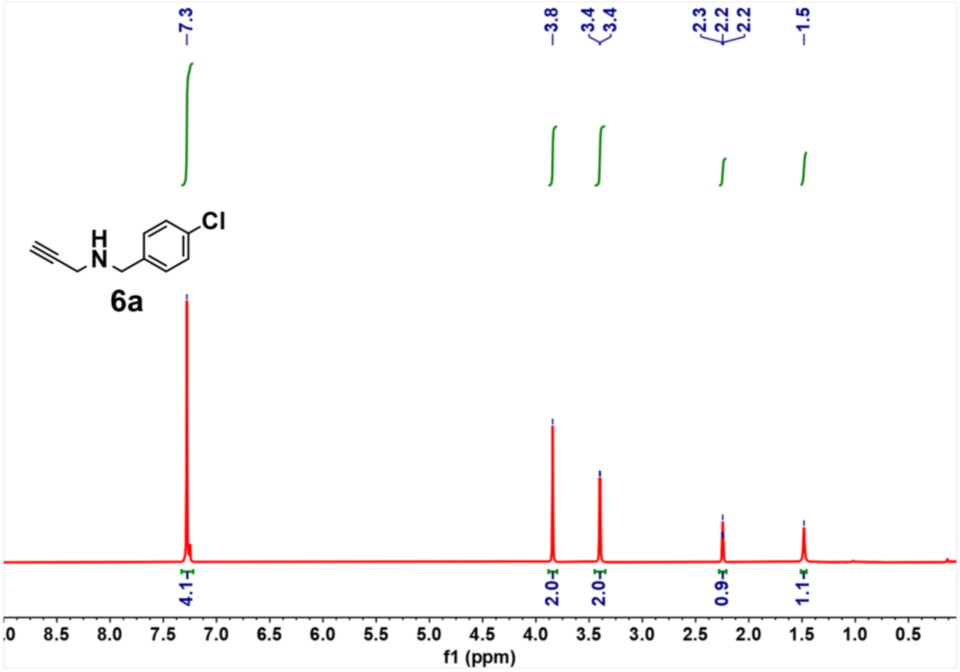

Supplementary Figure 31. ^{1}H NMR spectrum of (4-Chloro-benzyl)-prop-2-ynyl-amine. (in $CDCl_3$)

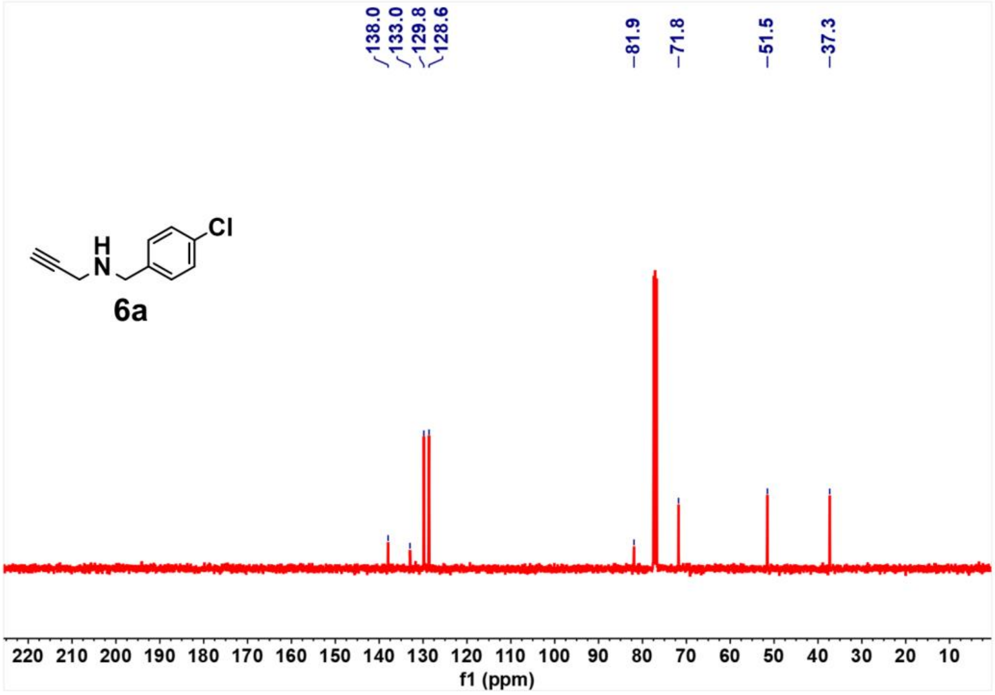

Supplementary Figure 32. ^{13}C NMR spectrum of (4-Chloro-benzyl)-prop-2-ynyl-amine. (in $CDCl_3$)

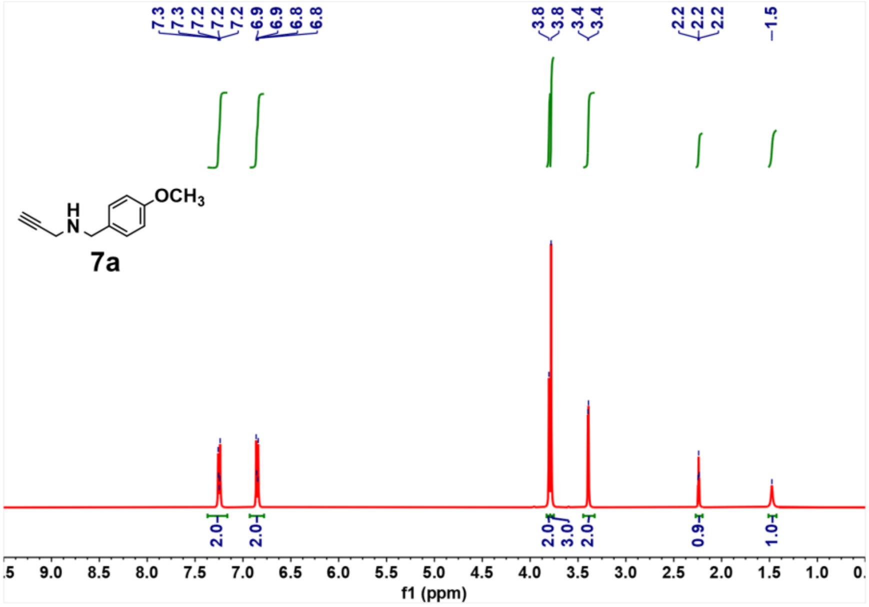

Supplementary Figure 33. ^{1}H NMR spectrum of (4-Methoxy-benzyl)-prop-2-ynyl-amine. (in $CDCl_3$)

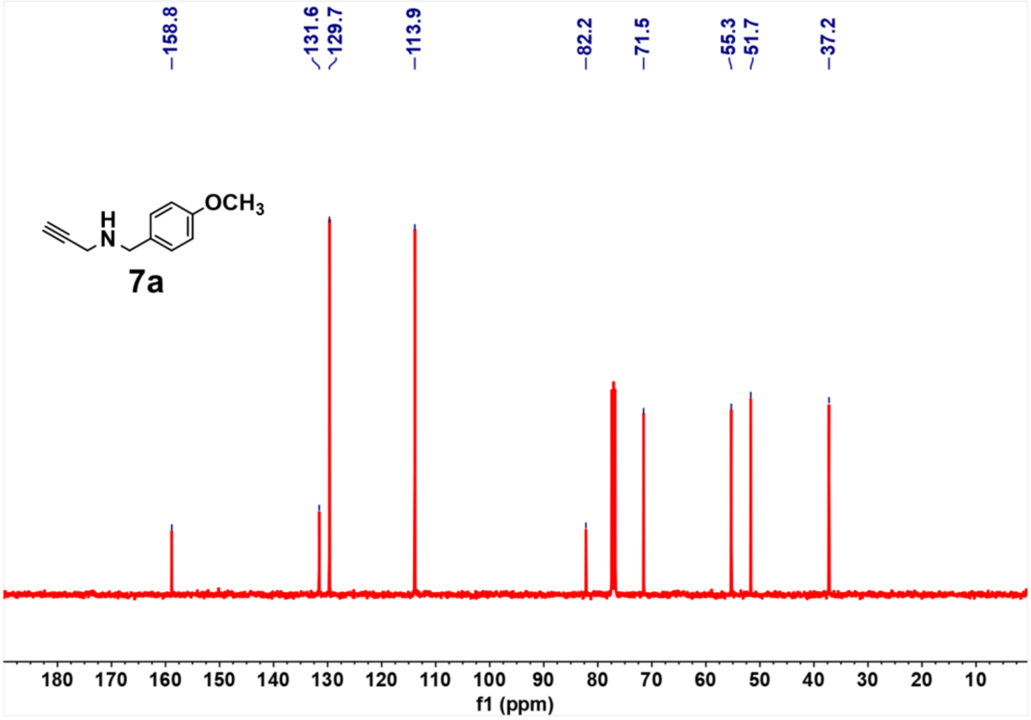

Supplementary Figure 34. ^{13}C NMR spectrum of (4-Methoxy-benzyl)-prop-2-ynyl-amine. (in $CDCl_3$)

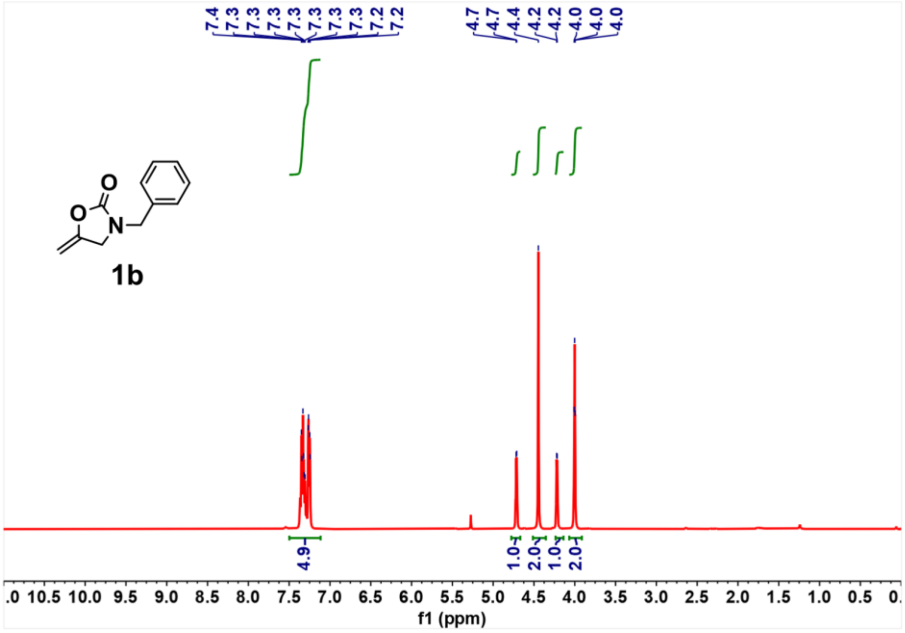

Supplementary Figure 35. ^{1}H NMR spectrum of 3-Benzyl-5-methylene-2-oxazolidinone. (in $CDCl_3$)

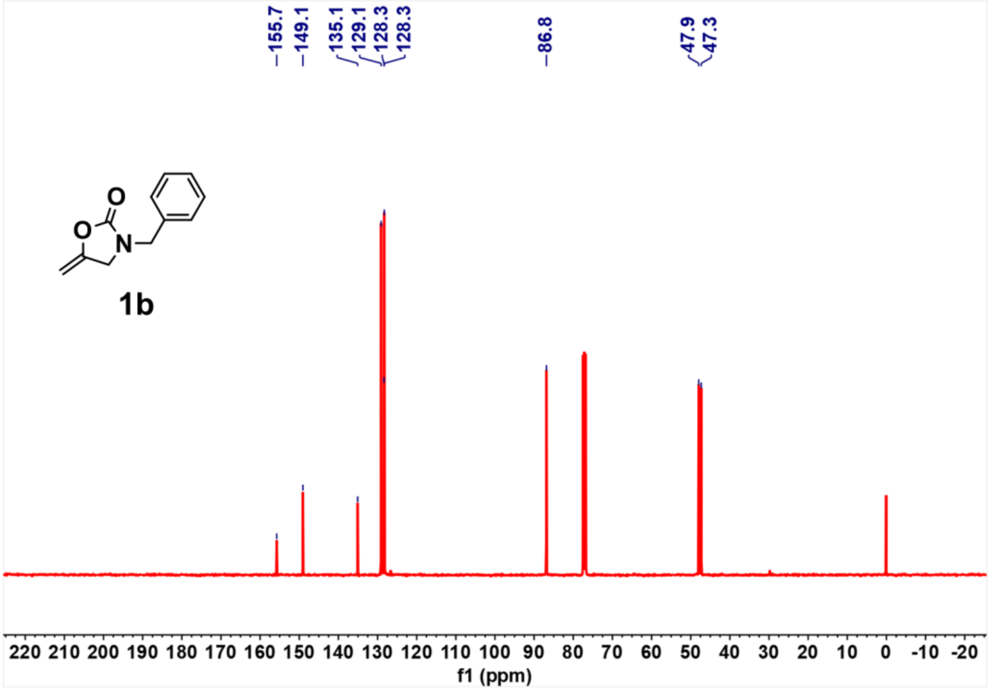

Supplementary Figure 36. ^{13}C NMR spectrum of 3-Benzyl-5-methylene-2-oxazolidinone(in $CDCl_3$)

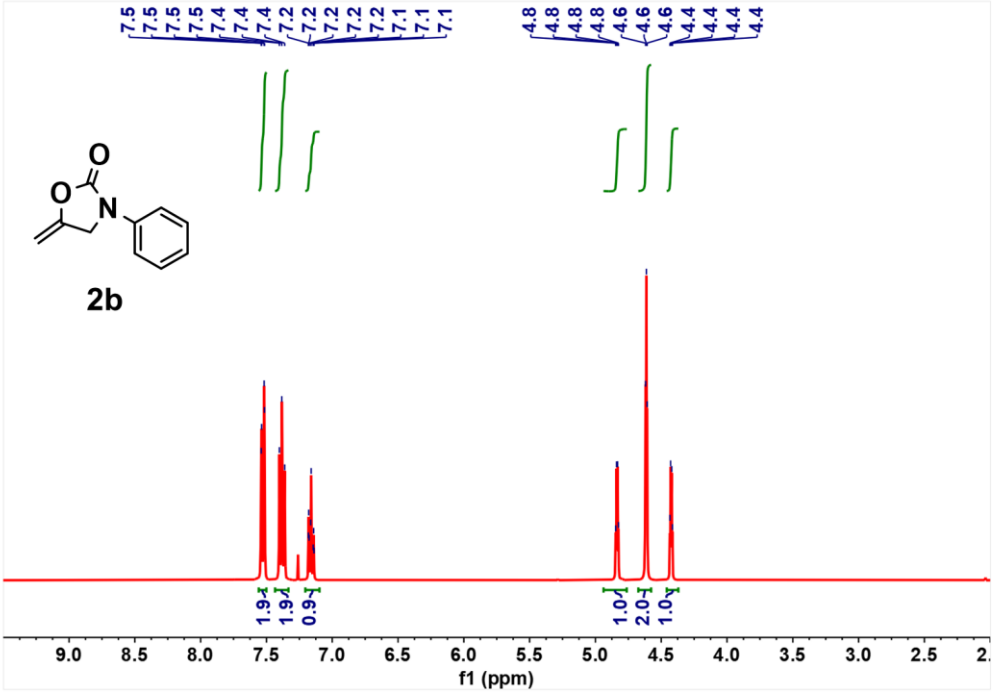

Supplementary Figure 37. ^{1}H NMR spectrum of 5-Methylene-3-phenyl-2-oxazolidinone. (in $CDCl_3$)

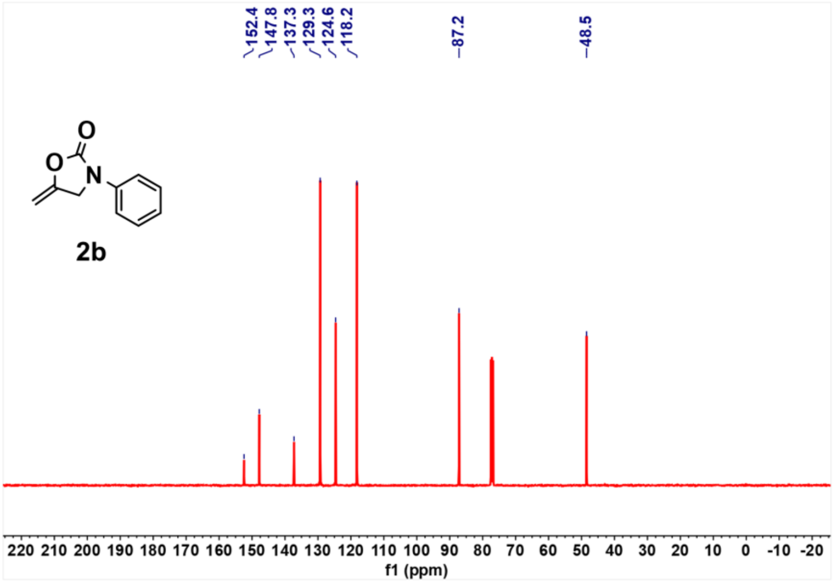

Supplementary Figure 38. ^{13}C NMR spectrum of 5-Methylene-3-phenyl-2-oxazolidinone. (in $CDCl_3$)

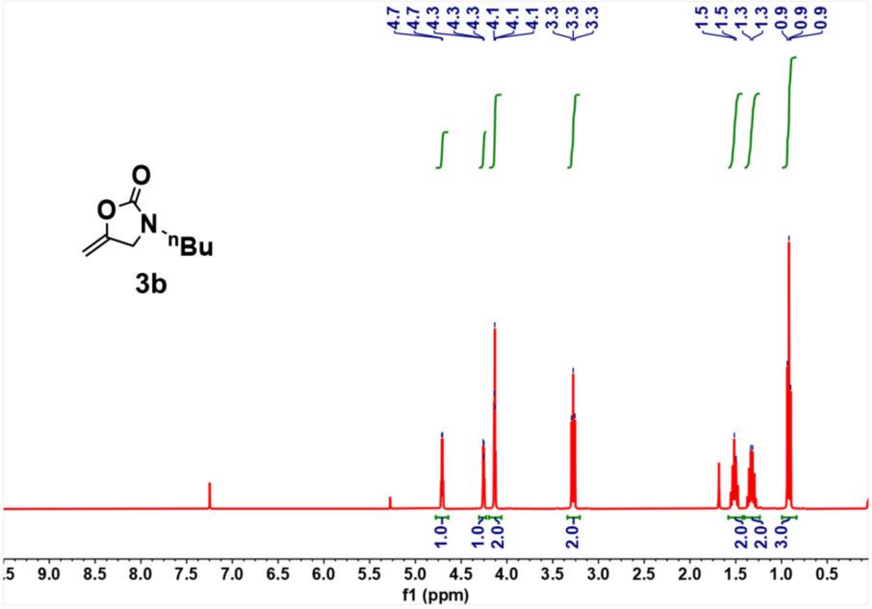

Supplementary Figure 39. ^{1}H NMR spectrum of 3-Butyl-5-methylene-2-oxazolidinone. (in $CDCl_3$)

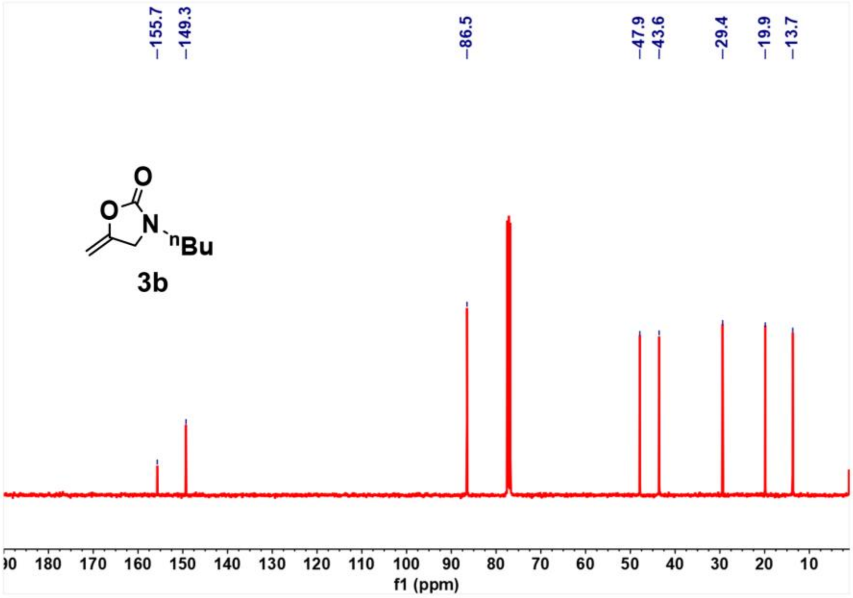

Supplementary Figure 40. ^{13}C NMR spectrum of 3-Butyl-5-methylene-2-oxazolidinone. (in $CDCl_3$)

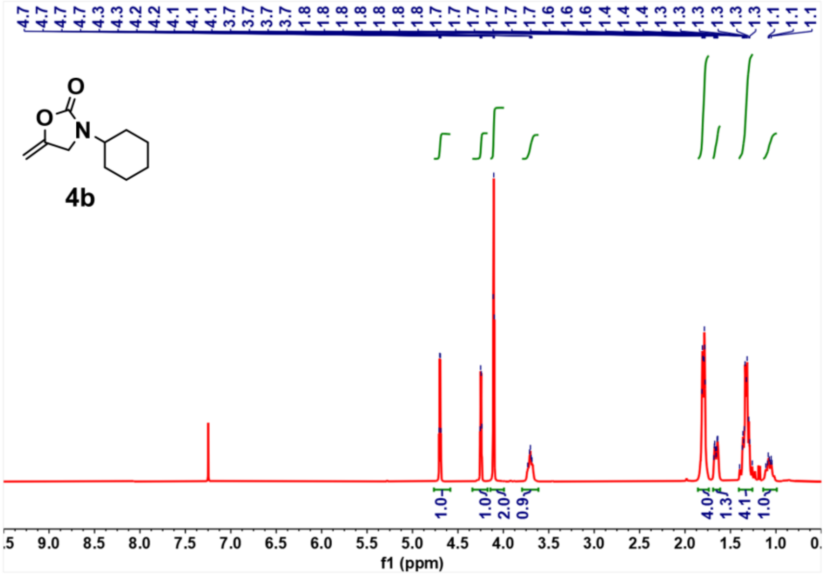

Supplementary Figure 41. ^1H NMR spectrum of N-Benzyl-2-propynylamine. (in $CDCl_3$)

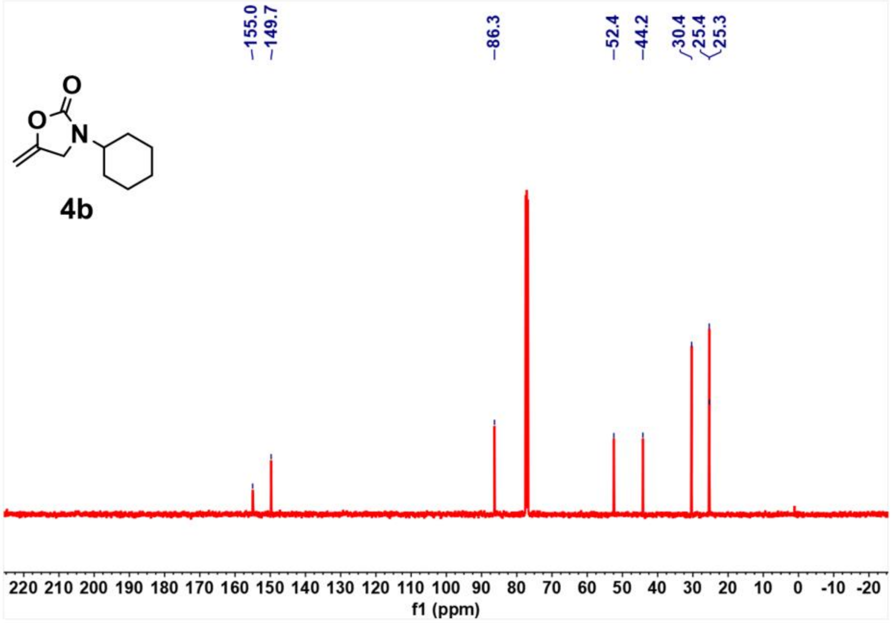

Supplementary Figure 42. ^{13}C NMR spectrum of N-Benzyl-2-propynylamine. (in $CDCl_3$)

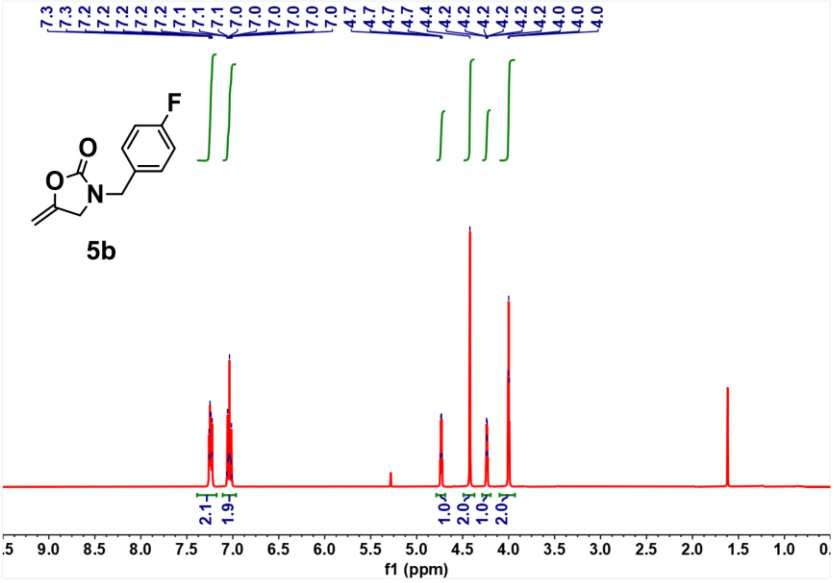

Supplementary Figure 43. ^{1}H NMR spectrum of 3-[(4-Fluorophenyl)methyl]-5-methylene-2-oxazolidinone. (in $CDCl_3$)

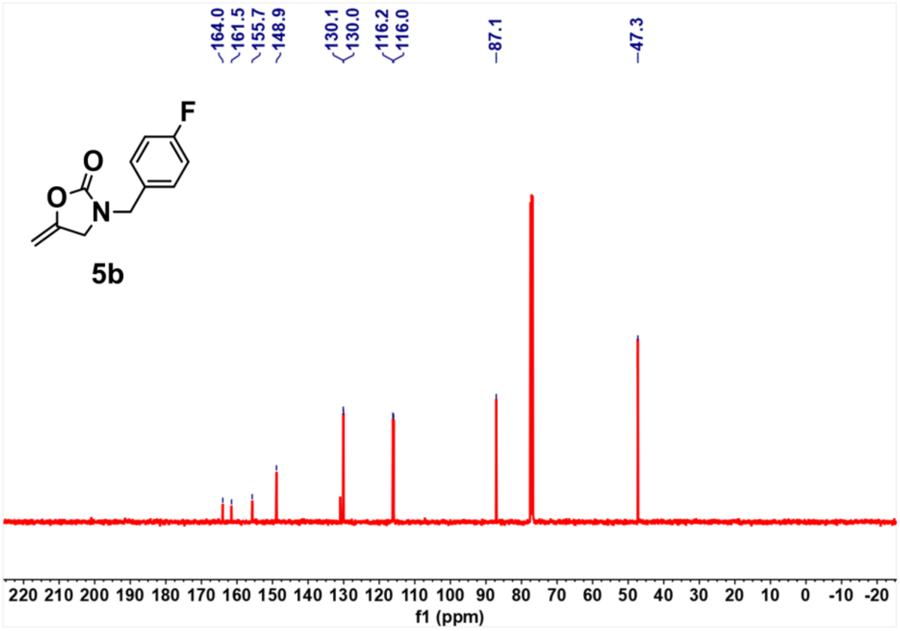

Supplementary Figure 44. ^{13}C NMR spectrum of 3-[(4-Fluorophenyl)methyl]-5-methylene-2-oxazolidinone. (in $CDCl_3$)

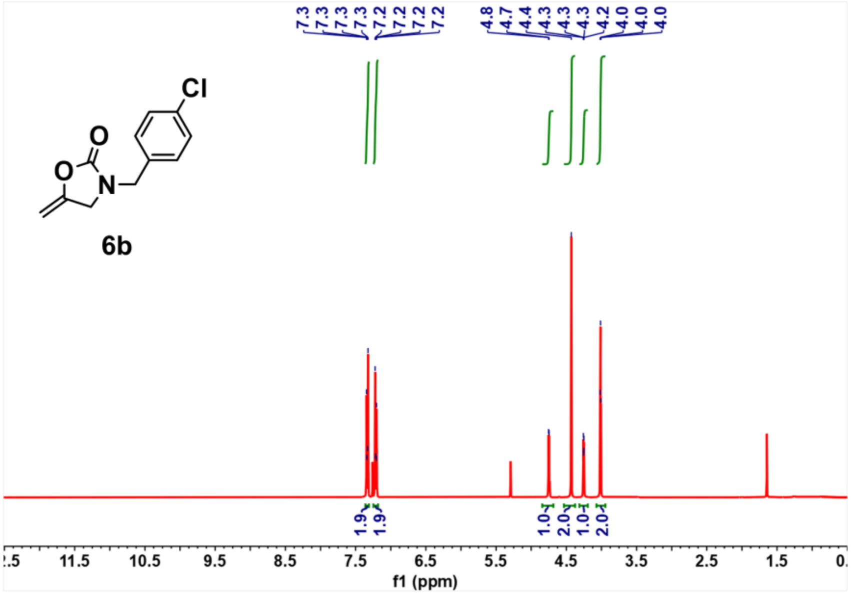

Supplementary Figure 45. ^{1}H NMR spectrum of 3-[(4-Chlorophenyl)methyl]-5-methylene-2-oxazolidinone. (in $CDCl_3$)

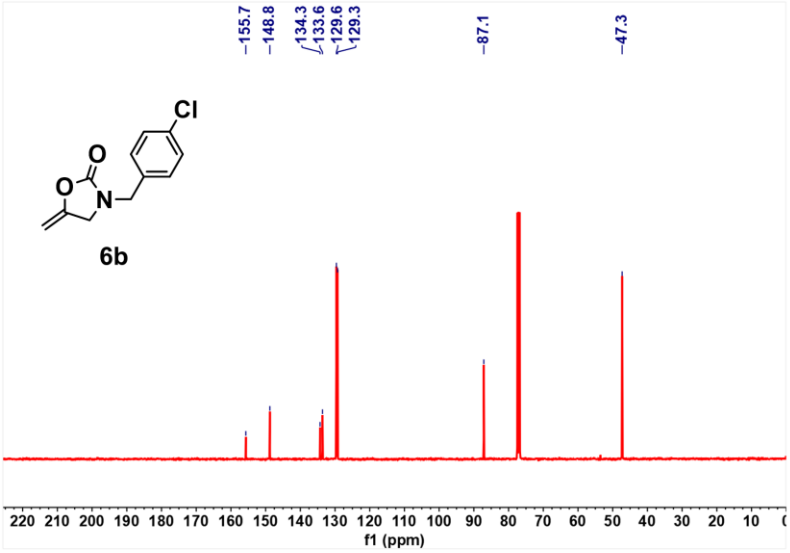

Supplementary Figure 46. ^{13}C NMR spectrum of 3-[(4-Chlorophenyl)methyl]-5-methylene-2-oxazolidinone. (in $CDCl_3$)

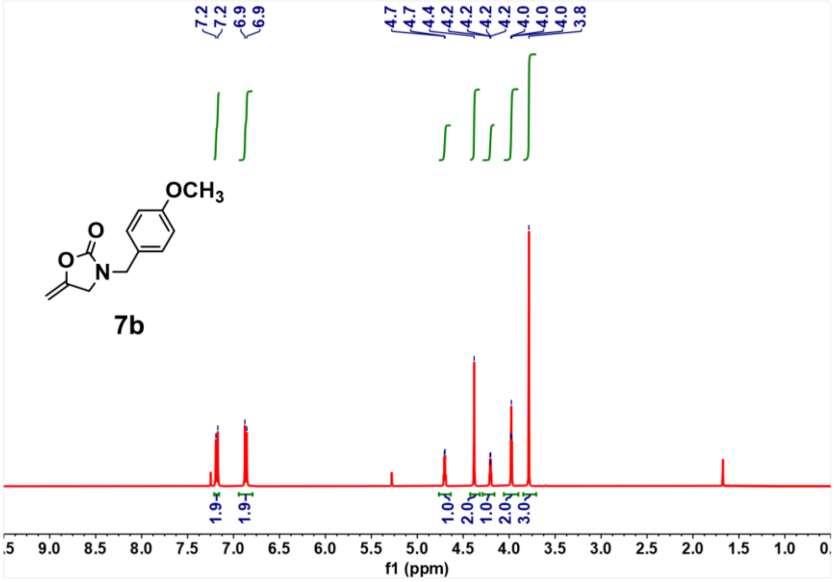

Supplementary Figure 47. ^{1}H NMR spectrum of 3-[(4-Methoxyphenyl)methyl]-5-methylene-2-oxazolidinone. (in $CDCl_3$)

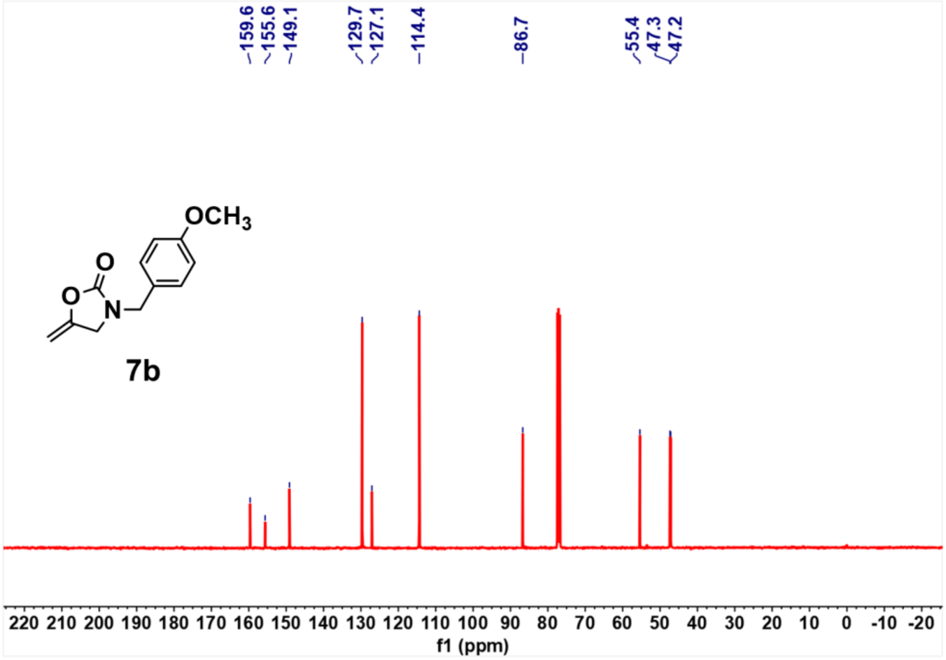

Supplementary Figure 48. ^{13}C NMR spectrum of 3-[(4-Methoxyphenyl)methyl]-5-methylene-2-oxazolidinone. (in $CDCl_3$)

Supplementary Tables

Supplementary Table 1: Cyclization of N-benzylprop-2-yn-1-amine with CO_2 by various solvents and catalysts.[a]

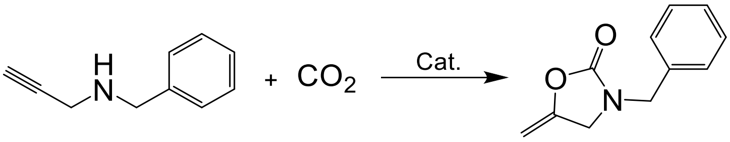

1a **1b**

Entry	Catalyst	Solvent	Base	*t* [h]	Yield[b] [%]
1	Ag_4	MeOH	DBU	2	27.80
2	Ag_4	THF	DBU	2	51.93
3	Ag_4	$CHCl_3$	DBU	2	95.90
4	Ag_4	MeCN	DBU	1.5	71.00
**5**	**Ag_4**	**MeCN**	**DBU**	**2**	**99.00**
6	Ag_4	MeCN	K_2CO_3	2	15.70
7[c]	Ag_2	MeCN	DBU	2	55.60
8[d]	Ag_6	MeCN	DBU	2	54.00
9[e]	Ag_9	MeCN	DBU	2	1.94
10[f]	$AgNO_3$	MeCN	DBU	2	32.17
11[g]	$AgBF_4$	MeCN	DBU	2	30.60
12[h]	$[Ag(C{\equiv}C^tBu)]_n$	MeCN	DBU	2	32.64
13[i]	Ag_4	MeCN	DBU	2	5.00
14[j]	DPPF	MeCN	DBU	2	0
15	none	MeCN	DBU	2	0

[a] Reaction conditions: catalyst (0.04 mol% based on Ag_4 NC), propargylamine (0.5 mmol), DBU (0.05 mmol), solvent (1 mL), 25℃ and CO_2 balloon. [b] Yields and selectivity were determined by gas chromatography. [c] $Ag_2(dppf)_3(BF_4)_2$ (0.06 mol%).[d] $[Ag_6H_4(dppm)_4(OAc)_2]$ (0.06 mol%). [e] $[Ag_9(1,2\text{-BDT})_6]^{3-}$ (0.05 mol%). [f] $AgNO_3$ (0.47 mol%). [g] $AgBF_4$ (0.4mol%). [h] $[Ag(C{\equiv}C^tBu)]_n$ (1.06 mol%). [i] without CO_2 balloon. [j] dppf (2 mg).

Supplementary Table 2: the cyclization of Various propargylamines with CO_2 [a].

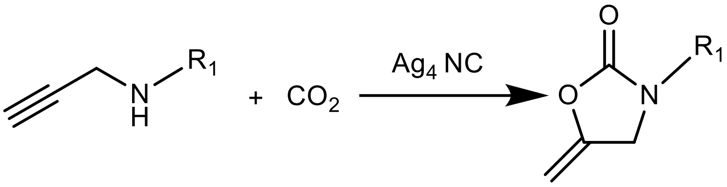

a b

Entry	Substrates	Products	Yield（%）
1	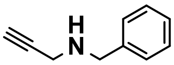 **1a**	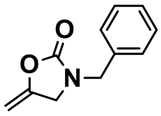 **1b**	99.0%
2[c]	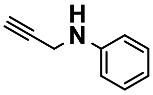 **2a**	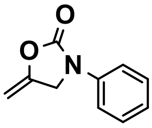 **2b**	87.0%
3	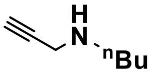 **3a**	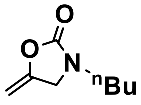 **3b**	98.2%
4	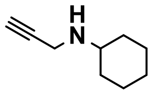 **4a**	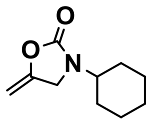 **4b**	99.0%
5	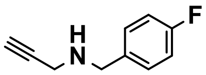 **5a**	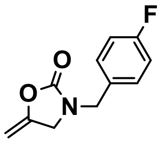 **5b**	85.7%
6	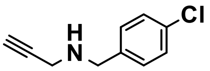 **6a**	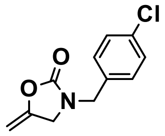 **6b**	93.0%
7	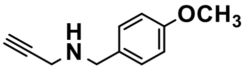 **7a**	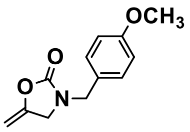 **7b**	92.9%

[a] Reaction conditions: catalyst (0.04 mol% based on Ag_4 NC), amine (0.5 mmol), DBU (0.05 mmol), solvent (1 mL), 2h, 25℃, and CO_2 balloon. [b] Yields and selectivity were determined by gas chromatography. [c] Reaction conditions: catalyst (0.04 mol% based on Ag_4 NC), N-Phenylpropargylamine (2a 0.5 mmol), DBU (0.05 mmol), solvent (1 mL), 12h, 25℃ and CO_2 balloon.

Supplementary Table 3. Different catalytic systems for the cyclization of Various propargylamines with CO_2.

Ent.	Catalyst	Loading (mol%)	T(℃)	Pressure (Mpa)	Time (h)	TON	TOF (h^{-1})	Ref.
1	$PrVO_4$/SnD	-	rt	1	7	-	-	10
2	[Zn_{116}]	0.27	70	0.1	12	367	30.6	11
3	Ag@Tppa-1	1.878	60	0.1	12	44.73	3.73	12
4	Ag@TpTa	1.878	60	0.1	10	51.65	5.17	12
5	Ag-rGO-3	6.7	30	0.1	12	13.43	1.12	13
6	Ag-MOF-1	3.98	rt	0.1	24	23.8	0.99	14
7	Bu_4NF	1	110	0.5	12	94	7.8	15
8	AgN@COF	-	55	0.1	10	120	12	16
9	KCC-1/IL/Ni@Pd NPs	0.1mg catalyst/1mol substrate	25	1	3	-	-	17
10	TMOF-3-Ag	10	rt	0.1	6	9.9	1.65	18
11	TOS-Ag_4	0.1	25	0.1	96	930	9.69	19
12	TNS-Ag_8	0.1	25	0.1	180	760	4.22	19
13	AuNP@PAMAM/C	1	40	1	10	99	9.9	20
14	$Cu_2H_{34}C_{36}N_{10}(PF_6)_2$	0.3	25-27	ballon	12	313	26.08	21
15	KCC/Salen/Ru(Ⅱ)	0.1mol catalyst/1mol substrate	100	1	1	-	-	22
16	[Au(dpb^F)]SbF_6	0.5	rt	Air	48	180	3.75	23
17	SiO_2/Fe_2O_3	-	120	2	20	-		24

Continuation of Supplementary Table 3.

Ent.	Catalyst	Loading (mol%)	T(℃)	Pressure(Mpa)	Time (h)	TON	TOF (h^{-1})	Ref.
18	$[Au_2(L)X_2]$	2	rt	0.1	24	49.5	2.06	25
19	$CoBr_2$[EEIM][OAC]	0.05	60	ballon	106	1740	16.4	26
20	Ag_{27}/MOF	0.025	25	0.1	240	1333	5.54	27
21	Ag@2,6-FPP-TAPT	0.052mol%	50	ballon	2	1928	964	28
22	Ag@3,5-FPP-TAPT	0.052mol%	50	ballon	2	1754	877	28
23	Ag@BT-COP	1.08 wt%	60	ballon	14	329.5	23.5	29
24	$[Mg_3Cu_2I_2(IN)_4(HCOO)_2(DEF)_4]_n$	10mg	25	0.1 MPa	6	68.24	22.75	30
25	Cu-TSP	1 mol%	50	ballon	24	99	4.15	31
26	CuBr@NH_2-MIL-101	15mol%	rt	ballon	8	6.6	0.825	32
27	Cu_2O@ZIF-8	8mol%	50	ballon	4	12.13	3.03	33
**28**	**Ag_4 NC**	**0.013mol % (One cluster as one active site)**	**25**	**ballon**	**2**	**5746.2**	**2873.1**	**This work**

Supplementary Reference:

1. Perdew, J. P. & Wang, Y. Accurate and simple analytic representation of the electron-gas correlation energy. *Physical Review B* **45**, 13244-13249 (1992).
2. Becke, A. D. Density‐functional thermochemistry. III. The role of exact exchange. *The Journal of Chemical Physics* **98**, 5648-5652 (1993).
3. Gaussian 09, Revision D.02, Frisch, M. J.; Trucks, G. W.; Schlegel, H. B.; Scuseria, G. E.; Robb, M. A.; Cheeseman, J. R.; Scalmani, G.; Barone, V.; Mennucci, B.; Petersson, G. A.; Nakatsuji, H.; Caricato, M.; Li, X.; Hratchian, H. P.; Izmaylov, A. F.;

Bloino, J.; Zheng, G.; Sonnenberg, J. L.; Hada, M.; Ehara, M.; Toyota, K.; Fukuda, R.; Hasegawa, J.; Ishida, M.; Nakajima, T.; Honda, Y.; Kitao, O.; Nakai, H.; Vreven, T.; Montgomery, Jr., J. A.; Peralta, J. E.; Ogliaro, F.; Bearpark, M.; Heyd, J. J.; Brothers, E.; Kudin, K. N.; Staroverov, V. N.; Kobayashi, R.; Normand, J.; Raghavachari, K.; Rendell, A.; Burant, J. C.; Iyengar, S. S.; Tomasi, J.; Cossi, M.; Rega, N.; Millam, J. M.; Klene, M.; Knox, J. E.; Cross, J. B.; Bakken, V.; Adamo, C.; Jaramillo, J.; Gomperts, R.; Stratmann, R. E.; Yazyev, O.; Austin, A. J.; Cammi, R.; Pomelli, C.; Ochterski, J. W.; Martin, R. L.; Morokuma, K.; Zakrzewski, V. G.; Voth, G. A.; Salvador, P.; Dannenberg, J. J.; Dapprich, S.; Daniels, A. D.; Farkas, O.; Foresman, J. B.; Ortiz, J. V.; Cioslowski, J.; Fox, D. J. Gaussian, Inc., Wallingford CT, 2009.

4. Ehlers, A. W. *et al.* A set of f-polarization functions for pseudo-potential basis sets of the transition metals Sc Cu, Y Ag and La Au. *Chemical Physics Letters* **208**, 111-114 (1993).
5. Hariharan, P. C. & Pople, J. A. The influence of polarization functions on molecular orbital hydrogenation energies. *Theoretica chimica acta* **28**, 213-222 (1973).
6. Marenich, A. V., Cramer, C. J. & Truhlar, D. G. Universal Solvation Model Based on Solute Electron Density and on a Continuum Model of the Solvent Defined by the Bulk Dielectric Constant and Atomic Surface Tensions. *The Journal of Physical Chemistry B* **113**, 6378-6396 (2009).
7. Grimme, S., Ehrlich, S. & Goerigk, L. Effect of the damping function in dispersion corrected density functional theory. *Journal of Computational Chemistry* **32**, 1456-1465 (2011).
8. Grimme, S., Antony, J., Ehrlich, S. & Krieg, H. A consistent and accurate ab initio parametrization of density functional dispersion correction (DFT-D) for the 94 elements H-Pu. *The Journal of Chemical Physics* **132**, 154104.
9. Brunel, P. *et al.* Valorization of CO_2: Preparation of 2-Oxazolidinones by Metal–Ligand Cooperative Catalysis with SCS Indenediide Pd Complexes. *ACS Catalysis* **7**, 2652-2660 (2017).
10. He, Z. *et al.* PrVO4/SnD NPs as a Nanocatalyst for Carbon Dioxide Fixation to

Synthesis Benzimidazoles and 2-Oxazolidinones. *Catalysis Letters* **151**, 1623-1632 (2021).

11. Cao, C. S. *et al.* Highly Efficient Conversion of Propargylic Amines and CO_2 Catalyzed by Noble-Metal-Free [Zn_{116}] Nanocages. *Angew Chem Int Ed Engl* **59**, 8586-8593 (2020).
12. Ghosh, S. *et al.* Utility of Silver Nanoparticles Embedded Covalent Organic Frameworks as Recyclable Catalysts for the Sustainable Synthesis of Cyclic Carbamates and 2-Oxazolidinones via Atmospheric Cyclizative CO_2 Capture. *ACS Sustainable Chemistry & Engineering* **8**, 5495-5513 (2020).
13. Zhang, X., Chen, K. H., Zhou, Z. H. & He, L. N. Reduced Graphene Oxide Supported Ag Nanoparticles: An Efficient Catalyst for CO_2 Conversion at Ambient Conditions. *ChemCatChem* **12**, 4825-4830, (2020).
14. Wang, X., Chang, Z., Jing, X., He, C. & Duan, C. Double-Helical Ag–S Rod-Based Porous Coordination Polymers with Double Activation: σ-Active and π-Active Functions. *ACS Omega* **4**, 10828-10833 (2019).
15. Fujii, A., Matsuo, H., Choi, J.-C., Fujitani, T. & Fujita, K.-i. Efficient synthesis of 2-oxazolidinones and quinazoline-2,4(1H,3H)-diones from CO2 catalyzed by tetrabutylammonium fluoride. *Tetrahedron* **74**, 2914-2920 (2018).
16. Islam, S. S., Biswas, S., Ali Molla, R., Yasmin, N. & Islam, S. M. Green Synthesized AgNPs Embedded in COF: An Efficient Catalyst for the Synthesis of 2 ‐ Oxazolidinones and α ‐ Alkylidene Cyclic Carbonates via CO_2 Fixation. *ChemNanoMat* **6**, 1386-1397 (2020).
17. Sadeghzadeh, S. M., Zhiani, R. & Emrani, S. Ni@Pd nanoparticles supported on ionic liquid-functionalized KCC-1 as robust and recyclable nanocatalysts for cycloaddition of propargylic amines and CO_2. *Applied Organometallic Chemistry* **32**, e3941 (2018).
18. Zhang, G., Yang, H. & Fei, H. Unusual Missing Linkers in an Organosulfonate-Based Primitive–Cubic (pcu)-Type Metal–Organic Framework for CO_2 Capture and Conversion under Ambient Conditions. *ACS Catalysis* **8**, 2519-2525 (2018).

19. Chang, Z., Jing, X., He, C., Liu, X. & Duan, C. Silver Clusters as Robust Nodes and π–Activation Sites for the Construction of Heterogeneous Catalysts for the Cycloaddition of Propargylamines. *ACS Catalysis* **8**, 1384-1391 (2018).

20. Matsuo, H., Fujii, A., Choi, J.-C., Fujitani, T. & Fujita, K.-i. Carboxylative Cyclization of Propargylic Amines with Carbon Dioxide- Catalyzed by Poly(amidoamine)-Dendrimer-Encapsulated Gold Nanoparticles. *Synlett* **30**, 1914-1918 (2019).

21. Chen, F. *et al.* Binuclear Tridentate Hemilabile Copper(I) Catalysts for Utilization of CO2 into Oxazolidinones from Propargylic Amines. *J Org Chem* **85**, 15197-15212 (2020).

22. Saadati, S. M. & Sadeghzadeh, S. M. KCC-1 Supported Ruthenium-Salen-Bridged Ionic Networks as a Reusable Catalyst for the Cycloaddition of Propargylic Amines and CO_2. *Catalysis Letters* **148**, 1692-1702, (2018).

23. Inagaki, F., Maeda, K., Nakazawa, K. & Mukai, C. Construction of the Oxazolidinone Framework from Propargylamine and CO_2 in Air at Ambient Temperature: Catalytic Effect of a Gold Complex Featuring an L_2/Z-Type Ligand. *European Journal of Organic Chemistry* **2018**, 2972-2976, doi:10.1002/ejoc.201800228 (2018).

24. Matsuo, H., Choi, J.-C., Fujitani, T. & Fujita, K.-i. Carboxylative Cyclization of a Propargylic Amine with CO_2 Catalyzed by a Silica-Coated Magnetite. *Chemical and Pharmaceutical Bulletin* **69**, 698-701, (2021).

25. A. C. A. Bayrakdar, T. *et al.* Dinuclear Gold(I) Complexes Bearing Alkyl-Bridged Bis(N-heterocyclic carbene) Ligands as Catalysts for Carboxylative Cyclization of Propargylamine: Synthesis, Structure, and Kinetic and Mechanistic Comparison to the Mononuclear Complex [Au^{IPr}Cl]. *Organometallics* **39**, 2907-2916 (2020).

26. Zhou, Z.-H., Chen, K.-H. & He, L.-N. Efficient and Recyclable Cobalt(II)/Ionic Liquid Catalytic System for CO_2 Conversion to Prepare 2-Oxazolinones at Atmospheric Pressure. *Chinese Journal of Chemistry* **37**, 1223-1228 (2019).

27. Zhao, M. *et al.* Ambient Chemical Fixation of CO2 Using a Robust Ag27 Cluster-Based Two-Dimensional Metal-Organic Framework. *Angew Chem Int Ed*

Engl **59**, 20031-20036 (2020).

28. Zhang, Y. *et al.* Controllable encapsulation of silver nanoparticles by porous pyridine-based covalent organic frameworks for efficient CO_2 conversion using propargylic amines. *Green Chemistry* **24**, 930-940, (2022).
29. Bai, X., Zhang, Y., Yan, F. & Lan, X. Thiadiazol-based conjugated organic polymer anchoring Ag nanoparticles for efficient conversion of CO_2 into oxazolidinones from propargylic amines. *Applied Surface Science* **604**, 154566 (2022).
30. Gu, A. L., Wang, W. T., Cheng, X. Y., Hu, T. D. & Wu, Z. L. Non-Noble-Metal Metal-Organic-Framework-Catalyzed Carboxylative Cyclization of Propargylic Amines with Atmospheric Carbon Dioxide under Ambient Conditions. *Inorg Chem* **60**, 13425-13433 (2021).
31. Xu, X., Li, Z., Huang, H., Jing, X. & Duan, C. A novel copper metal–organic framework catalyst for the highly efficient conversion of CO_2 with propargylic amines. *Inorganic Chemistry Frontiers* **9**, 3839-3844 (2022).
32. Cui, H.-Y., Zhang, Y.-X., Cao, C.-S., Hu, T.-D. & Wu, Z.-L. Engineering noble-metal-free metal–organic framework composite catalyst for efficient CO_2 conversion under ambient conditions. *Chemical Engineering Journal* **451**, 138764 (2023).
33. Gu, A.-L. *et al.* Highly Efficient Conversion of Propargylic Alcohols and Propargylic Amines with CO_2 Activated by Noble-Metal-Free Catalyst Cu_2O@ZIF-8. *Angewandte Chemie International Edition* **61**, e202114817 (2022).
